# Tuning Transcriptional Regulation through Signaling: A Predictive Theory of Allosteric Induction

**DOI:** 10.1016/j.cels.2018.02.004

**Published:** 2018-03-21

**Authors:** Manuel Razo-Mejia, Stephanie L. Barnes, Nathan M. Belliveau, Griffin Chure, Tal Einav, Mitchell Lewis, Rob Phillips

**Affiliations:** 1 Division of Biology and Biological Engineering, California Institute of Technology, Pasadena, CA 91125, USA; 2 Department of Physics, California Institute of Technology, Pasadena, CA 91125, USA; 3 Department of Biochemistry and Biophysics, University of Pennsylvania School of Medicine, Philadelphia, PA 19104, USA; 4 Department of Applied Physics, California Institute of Technology, Pasadena, CA 91125, USA

## Abstract

Allosteric regulation is found across all domains of life, yet we still lack simple, predictive theories that directly link the experimentally tunable parameters of a system to its input-output response. To that end, we present a general theory of allosteric transcriptional regulation using the Monod-Wyman-Changeux model. We rigorously test this model using the ubiquitous simple repression motif in bacteria by first predicting the behavior of strains that span a large range of repressor copy numbers and DNA binding strengths and then constructing and measuring their response. Our model not only accurately captures the induction profiles of these strains, but also enables us to derive analytic expressions for key properties such as the dynamic range and [EC_50_]. Finally, we derive an expression for the free energy of allosteric repressors that enables us to collapse our experimental data onto a single master curve that captures the diverse phenomenology of the induction profiles.

## In Brief

Allosteric regulation is found across all domains of life, yet we still lack simple, predictive theories that link the experimentally tunable parameters of such systems to their input-output response. We present a general theory of allosteric transcriptional regulation that is rigorously tested using a well-characterized regulatory system in bacteria. Our model not only accurately captures our data, but also enables us to derive analytic expressions for key phenotypic properties and is broadly applicable to other regulatory systems in bacteria.

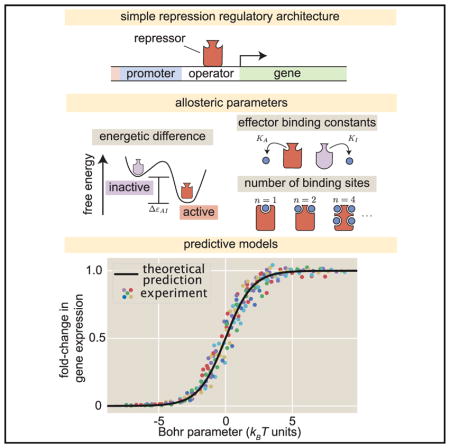

## INTRODUCTION

Understanding how organisms sense and respond to changes in their environment has long been a central theme of biological inquiry. At the cellular level, this interaction is mediated by a diverse collection of molecular signaling pathways. A pervasive mechanism of signaling in these pathways is allosteric regulation, in which the binding of a ligand induces a conformational change in some target molecule, triggering a signaling cascade (Lindsley and Rutter, 2006). One of the most important examples of such signaling is offered by transcriptional regulation, whereby a transcription factor’s propensity to bind to DNA will be altered upon binding to an allosteric effector.

Despite allostery’s ubiquity, we lack a formal, rigorous, and generalizable framework for studying its effects across the broad variety of contexts in which it appears. A key example of this is transcriptional regulation, in which allosteric transcription factors can be induced or corepressed by binding to a ligand. An allosteric transcription factor can adopt multiple conformational states, each of which has its own affinity for the ligand and for its DNA target site. *In vitro* studies have rigorously quantified the equilibria of different conformational states for allosteric transcription factors and measured the affinities of these states to the ligand (Harman, 2001; Lanfranco et al., 2017). Despite these experimental observations, the lack of a coherent quantitative model for allosteric transcriptional regulation has made it impossible to predict the behavior of even a simple genetic circuit across a range of regulatory parameters.

The ability to predict circuit behavior robustly—that is, across both broad ranges of parameters and regulatory architectures—is important for multiple reasons. First, in the context of a specific gene, accurate prediction demonstrates that all components relevant to the gene’s behavior have been identified and characterized to sufficient quantitative precision. Second, in the context of genetic circuits in general, robust prediction validates the model that generated the prediction. Possessing a validated model also has implications for future work. For example, when we have sufficient confidence in the model, a single dataset can be used to accurately extrapolate a system’s behavior in other conditions. Moreover, there is an essential distinction between a predictive model, which is used to predict a system’s behavior given a set of input variables, and a retroactive model, which is used to describe the behavior of data that has already been obtained. We note that even some of the most careful and rigorous analysis of transcriptional regulation often entails only a retroactive reflection on a single experiment. This raises the fear that each regulatory architecture may require a unique analysis that cannot carry over to other systems, a worry that is exacerbated by the prevalent use of phenomenological functions (e.g., Hill functions or ratios of polynomials) that can analyze a single dataset but cannot be used to extrapolate a system’s behavior in other conditions (Setty et al., 2003; Poelwijk et al., 2011; Vilar and Saiz, 2013; Rogers et al., 2015; Rohlhill et al., 2017).

**Figure 1:**
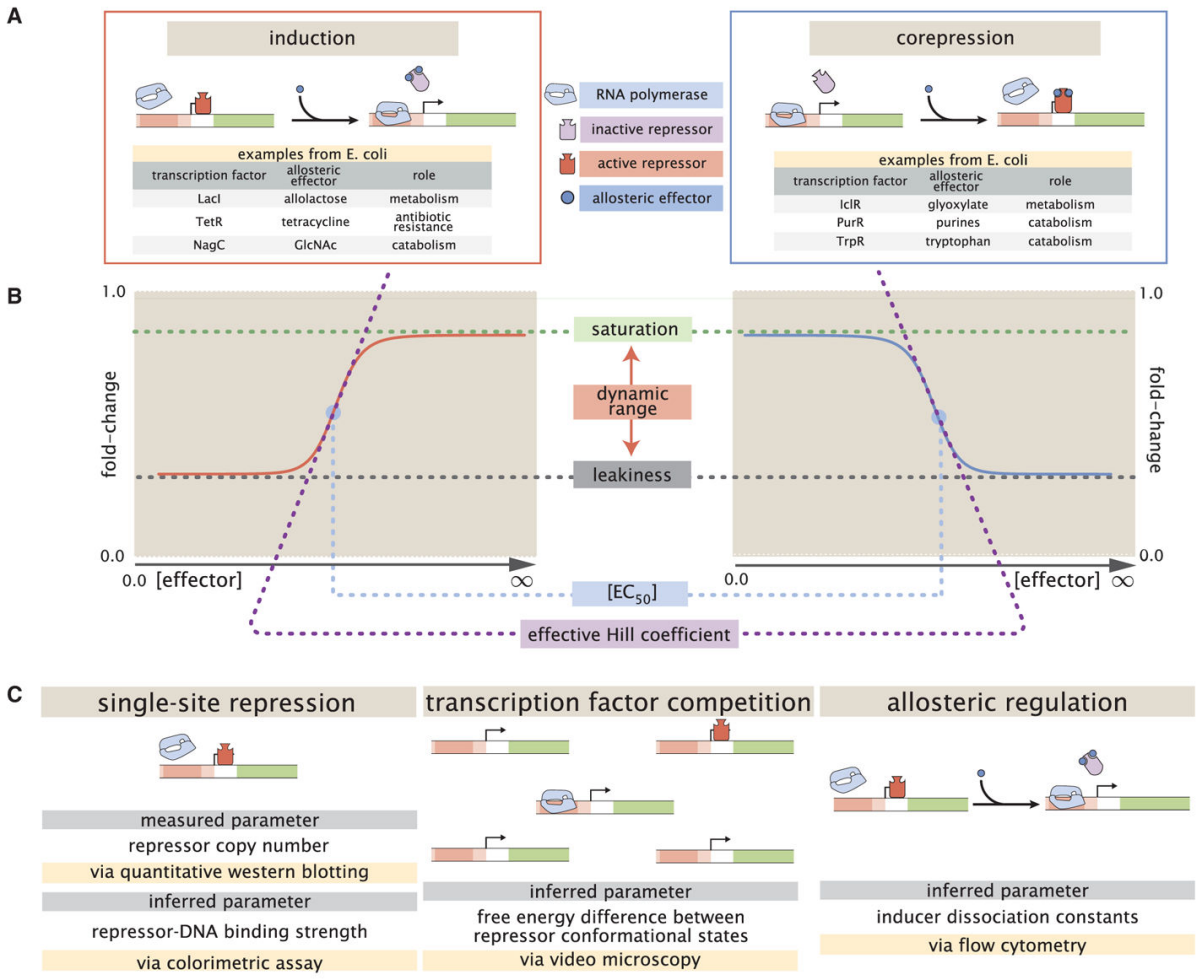
Transcription Regulation Architectures Involving an Allosteric Repressor (A) We consider a promoter regulated solely by an allosteric repressor. When bound, the repressor prevents RNAP from binding and initiating transcription. Induction is characterized by the addition of an effector that binds to the repressor and stabilizes the inactive state (defined as the state with a low affinity for DNA), thereby increasing gene expression. In corepression, the effector stabilizes the repressor’s active state and thus further reduces gene expression. We list several characterized examples of induction and corepression that support different physiological roles in *E. coli* (Huang et al., 2011; Li et al., 2014). (B) A schematic regulatory response of the two architectures shown in (A) plotting the fold-change in gene expression as a function of effector concentration, where fold-change is defined as the ratio of gene expression in the presence versus the absence of repressor. We consider the following key phenotypic properties that describe each response curve: the minimum response (leakiness), the maximum response (saturation), the difference between the maximum and minimum response (dynamic range), the concentration of ligand that generates a fold-change halfway between the minimal and maximal response ([EC_50_]), and the log-log slope at the midpoint of the response (effective Hill coefficient). (C) Over time, we have refined our understanding of simple repression architectures. A first round of experiments used colorimetric assays and quantitative western blots to investigate how single-site repression is modified by the repressor copy number and repressor-DNA binding energy (Garcia and Phillips, 2011). A second round of experiments used video microscopy to probe how the copy number of the promoter and presence of competing repressor binding sites affect gene expression, and we use this dataset to determine the free energy difference between the repressor’s inactive and active conformations (Weinert et al., 2014). Here we used flow cytometry to determine the inducer-repressor dissociation constants and demonstrate that with these parameters we can predict a priori the behavior of the system for any repressor copy number, DNA binding energy, gene copy number, and inducer concentration.

This work explores what happens when theory takes center stage, namely, when we first write down the equations governing a system and describe its expected behavior across a wide array of experimental conditions, and only then do we set out to experimentally confirm these results. Building upon previous work (Garcia and Phillips, 2011; Brewster et al., 2014; Weinert et al., 2014) and the work of Monod, Wyman, and Changeux (Monod et al., 1965), we present a statistical mechanical rendering of allostery in the context of induction and corepression (shown schematically in Figure 1A, henceforth referred to as the MWC model) and use it as the basis of parameter-free predictions, which we then test experimentally. More specifically, we study the simple repression motif—a widespread bacterial genetic regulatory architecture in which binding of a transcription factor occludes binding of an RNA polymerase, thereby inhibiting transcription initiation. The MWC model stipulates that an allosteric protein fluctuates between two distinct conformations, an active and an inactive state, in thermodynamic equilibrium (Monod et al., 1965). During induction, for example, effector binding increases the probability that a repressor will be in the inactive state, weakening its ability to bind to the promoter and resulting in increased expression. To test the predictions of our model across a wide range of operator binding strengths and repressor copy numbers, we design an *Escherichia coli* genetic construct in which the binding probability of a repressor regulates gene expression of a fluorescent reporter.

In total, the work presented here demonstrates that one extremely compact set of parameters can be applied self-consistently and predictively to different regulatory situations including simple repression on the chromosome, cases in which decoy binding sites for repressor are put on plasmids, cases in which multiple genes compete for the same regulatory machinery, cases involving multiple binding sites for repressor leading to DNA looping, and induction by signaling (Garcia and Phillips, 2011; Garcia et al., 2011; Brewster et al., 2012, 2014; Boedicker et al., 2013a, 2013b). Thus, rather than viewing the behavior of each circuit as giving rise to its own unique input-output response, the MWC model provides a means to characterize these seemingly diverse behaviors using a single unified framework governed by a small set of parameters.

## RESULTS

### Characterizing Transcription Factor Induction Using the Monod-Wyman-Changeux Model

We begin by considering a simple repression genetic architecture in which the binding of an allosteric repressor occludes the binding of RNA polymerase (RNAP) to the DNA (Ackers et al., 1982; Buchler et al., 2003). When an effector (hereafter referred to as an “inducer” for the case of induction) binds to the repressor, it shifts the repressor’s allosteric equilibrium toward the inactive state as specified by the MWC model (Monod et al., 1965). This causes the repressor to bind more weakly to the operator, which increases gene expression. Simple repression motifs in the absence of inducer have been previously characterized by an equilibrium model in which the probability of each state of repressor and RNAP promoter occupancy is dictated by the Boltzmann distribution (Ackers et al., 1982; Buchler et al., 2003; Vilar and Leibler, 2003; Bintu et al., 2005a; Garcia and Phillips, 2011; Brewster et al., 2014) (we note that non-equilibrium models of simple repression have been shown to have the same functional form that we derive below; Phillips, 2015). We extend these models to consider allostery by accounting for the equilibrium state of the repressor through the MWC model.

**Figure 2:**
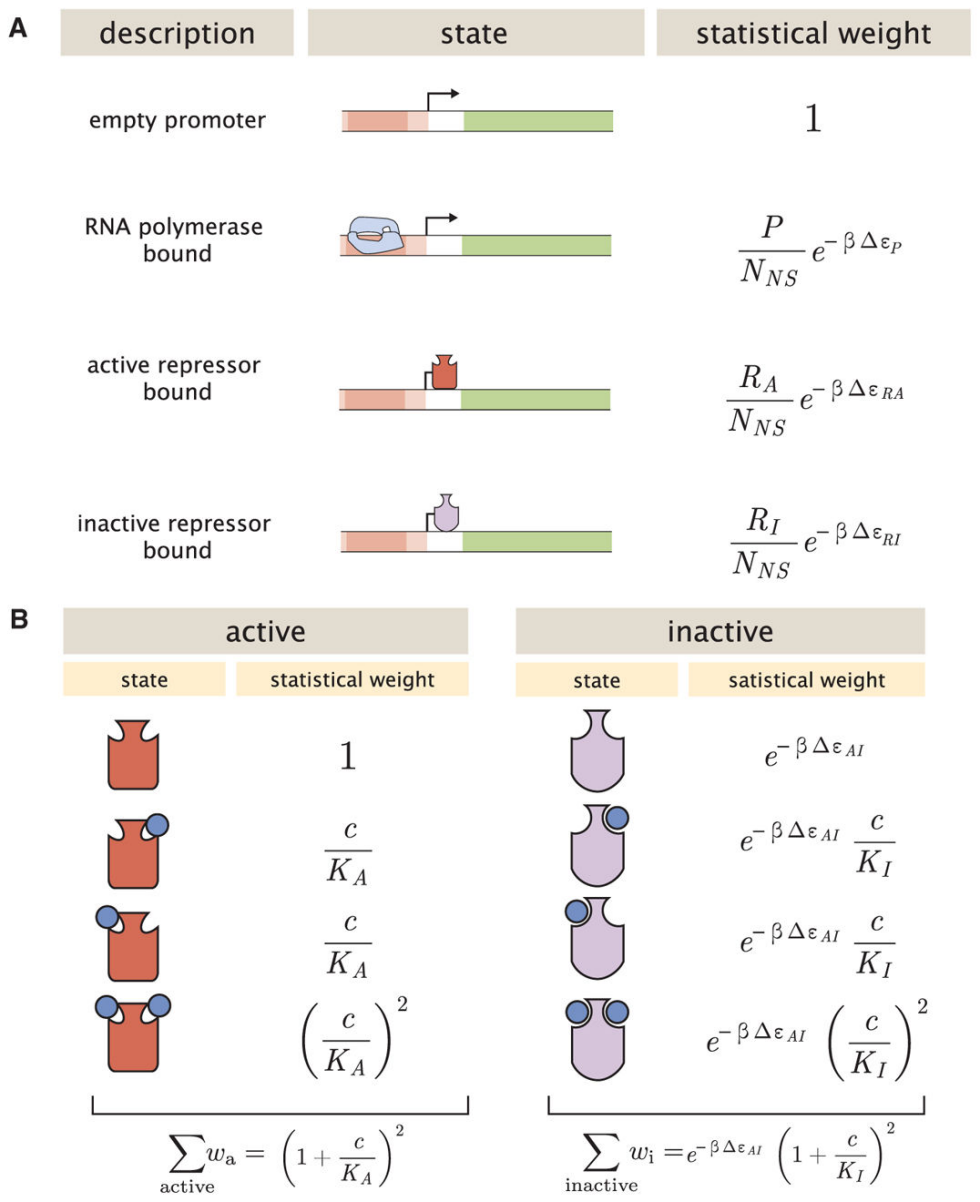
States and Weights for the Simple Repression Motif (A) RNAP (light blue) and a repressor compete for binding to a promoter of interest. There are *R_A_* repressors in the active state (red) and *R_I_* repressors in the inactive state (purple). The difference in energy between a repressor bound to the promoter of interest versus another non-specific site elsewhere on the DNA equals Δε*_RA_* in the active state and Δε*_RI_* in the inactive state; the *P* RNAP have a corresponding energy difference Δε*_P_* relative to nonspecific binding on the DNA. *N_NS_* represents the number of non-specific binding sites for both RNAP and repressor. (B) A repressor has an active conformation (red, left column) and an inactive conformation (purple, right column), with the energy difference between these two states given by Δε*_AI_*. The inducer (blue circle) at concentration *c* is capable of binding to the repressor with dissociation constants *K_A_* in the active state and *K_I_* in the inactive state. The eight states for a dimer with *n* = 2 inducer binding sites are shown along with the sums of the statistical weights of the active and inactive states.

Thermodynamic models of gene expression begin by enumerating all possible states of the promoter and their corresponding statistical weights. As shown in Figure 2A, the promoter can either be empty, occupied by RNAP, or occupied by either an active or an inactive repressor. The probability that RNAP binds to the promoter depends upon the protein copy numbers, which we denote as *P* for RNAP, *R_A_* for active repressor, and *R_I_* for inactive repressor. We note that repressors fluctuate between the active and inactive conformation in thermodynamic equilibrium, such that *R_A_* and *R_I_* will remain constant for a given inducer concentration (Monod et al., 1965). We assign the repressor a different DNA binding affinity in the active and inactive state. In addition to the specific binding sites at the promoter, we assume that there are *N_NS_* non-specific binding sites elsewhere (i.e., on parts of the genome outside the simple repression architecture) where the RNAP or the repressor can bind. All specific binding energies are measured relative to the average non-specific binding energy. Thus, Δε*_P_* represents the energy difference between the specific and non-specific binding for RNAP to the DNA. Likewise, Δε*_RA_* and Δε*_RI_* represent the difference in specific and nonspecific binding energies for repressor in the active or inactive state, respectively.

Thermodynamic models of transcription (Ackers et al., 1982; Buchler et al., 2003; Vilar and Leibler, 2003; Bintu et al., 2005a, 2005b; Kuhlman et al., 2007; Daber et al., 2011; Garcia and Phillips, 2011; Brewster et al., 2014; Weinert et al., 2014) posit that gene expression is proportional to the probability that the RNAP is bound to the promoter *p*_bound_, which is given by

(Equation 1) $$p_{\text{bound}}=\frac{\frac{P}{N_{NS}}e^{-\beta \Delta \varepsilon_{P}}}{1+\frac{R_{A}}{N_{NS}}e^{-\beta \Delta \varepsilon_{RA}}+\frac{R_{I}}{N_{NS}}e^{-\beta \Delta \varepsilon_{RI}}+\frac{P}{N_{NS}}e^{-\beta \Delta \varepsilon_{P}}},$$

with $\beta =\frac{1}{k_{B}T}$ where *k_B_* is the Boltzmann constant and *T* is the temperature of the system. As *k_B_T* is the natural unit of energy at the molecular length scale, we treat the products *β*Δε*_j_* as single parameters within our model. Measuring *p*_bound_ directly is fraught with experimental difficulties, as determining the exact proportionality between expression and *p*_bound_ is not straightforward. Instead, we measure the fold-change in gene expression due to the presence of the repressor. We define fold-change as the ratio of gene expression in the presence of repressor relative to expression in the absence of repressor (i.e., constitutive expression), namely,

(Equation 2) $$\text{fold-change}\equiv \frac{p_{\text{bound}}(R>0)}{p_{\text{bound}}(R=0)}.$$

We can simplify this expression using two well-justified approximations: (1) $\frac{P}{N_{NS}}e^{-\beta \Delta \varepsilon_{P}}\ll 1$, implying that the RNAP binds weakly to the promoter (*N_NS_* = 4.6 × 10^6^, *P* ≈ 10^3^ (Klumpp and Hwa, 2008), Δ*ε_P_* ≈ −2 to −5 *k_B_T* (Brewster et al., 2012), so that $\frac{P}{N_{NS}}e^{-\beta \Delta \varepsilon_{P}}\approx 0.01$) and (2) $\frac{R_{I}}{N_{NS}}e^{-\beta \Delta \varepsilon_{RI}}\ll 1+\frac{R_{A}}{N_{NS}}e^{-\beta \Delta \varepsilon_{RA}}$, which reflects our assumption that the inactive repressor binds weakly to the promoter of interest. Using these approximations, the fold-change reduces to the form

(Equation 3) $$\text{fold-change}\approx {\left(1+\frac{R_{A}}{N_{NS}}e^{-\beta \Delta \varepsilon_{RA}}\right)}^{-1}\equiv {\left(1+p_{A}(c)\frac{R}{N_{NS}}e^{-\beta \Delta \varepsilon_{RA}}\right)}^{-1},$$

where in the last step we have introduced the fraction *p_A_*(*c*) of repressors in the active state given a concentration *c* of inducer, such that *R_A_*(*c*) = *p_A_*(*c*)*R*. Since inducer binding shifts the repressors from the active to the inactive state, *p_A_*(*c*) grows smaller as *c* increases (Marzen et al., 2013).

We use the MWC model to compute the probability *p_A_*(*c*) that a repressor with *n* inducer binding sites will be active. The value of *p_A_*(*c*) is given by the sum of the weights of the active repressor states divided by the sum of the weights of all possible repressor states (see Figure 2B), namely,

(Equation 4) $$p_{A}(c)=\frac{{\left(1+\frac{c}{K_{A}}\right)}^{n}}{{\left(1+\frac{c}{K_{A}}\right)}^{n}+e^{-\beta \Delta \varepsilon_{AI}}\;{\left(1+\frac{c}{K_{I}}\right)}^{n}},$$

where *K_A_* and *K_I_* represent the dissociation constant between the inducer and repressor in the active and inactive states, respectively, and Δε*_AI_* = ε*_I_* − ε*_A_* is the free energy difference between a repressor in the inactive and active state (the quantity *e*^−Δ*ε*_*AI*_^ is sometimes denoted by *L* [Monod et al., 1965; Marzen et al., 2013] or *K*_RR*_ [Daber et al., 2011]). In this equation, $\frac{c}{K_{A}}$ and $\frac{c}{K_{I}}$ represent the change in free energy when an inducer binds to a repressor in the active or inactive state, respectively, while *e*^−*β*Δ*ε*_*AI*_^ represents the change in free energy when the repressor changes from the active to inactive state in the absence of inducer. Thus, a repressor that favors the active state in the absence of inducer (Δε*_AI_* > 0) will be driven toward the inactive state upon inducer binding when *K_I_* < *K_A_*. The specific case of a repressor dimer with *n* = 2 inducer binding sites is shown in Figure 2B.

Substituting *p_A_*(*c*) from Equation 4 into Equation 3 yields the general formula for induction of a simple repression regulatory architecture (Phillips, 2015), namely,

(Equation 5) $$\text{fold-change}={\left(1+\frac{{\left(1+\frac{c}{K_{A}}\right)}^{n}}{{\left(1+\frac{c}{K_{A}}\right)}^{n}+e^{-\beta \Delta \varepsilon_{AI}}\;{\left(1+\frac{c}{K_{I}}\right)}^{n}}\frac{R}{N_{NS}}e^{-\beta \Delta \varepsilon_{RA}}\right)}^{-1}.$$

While we have used the specific case of simple repression with induction to craft this model, the same mathematics describe the case of corepression in which binding of an allosteric effector stabilizes the active state of the repressor and decreases gene expression (see Figure 1B). A notable property of this model is that we shift from induction (governed by *K_I_* < *K_A_*) to corepression (*K_I_* > *K_A_*) as the ligand transitions from preferentially binding to the inactive repressor state to stabilizing the active state. Furthermore, this general approach can be used to describe a variety of other motifs such as activation, multiple repressor binding sites, and combinations of activator and repressor binding sites (Bintu et al., 2005b; Brewster et al., 2014; Weinert et al., 2014).

The formula presented in Equation 5 enables us to make precise quantitative statements about induction profiles. Motivated by the broad range of predictions implied by Equation 5, we designed a series of experiments using the *lac* system in *E. coli* to tune the control parameters for a simple repression genetic circuit. As discussed in Figure 1C, previous studies from our lab have provided well-characterized values for many of the parameters in our experimental system, leaving only the values of the MWC parameters (*K_A_*, *K_I_*, and Δε*_AI_*) to be determined. We note that while previous studies have obtained values for *K_A_*, *K_I_*, and *L* = *e*^−*β*Δ*ε*_*AI*_^ (O’Gorman et al., 1980; Daber et al., 2011), they were either based upon biochemical experiments or *in vivo* conditions involving poorly characterized transcription factor copy numbers and gene copy numbers. These differences relative to our experimental conditions and fitting techniques led us to believe that it was important to perform our own analysis of these parameters. After inferring these three MWC parameters (see STAR Methods section “Inferring Allosteric Parameters from Previous Data” for details regarding the inference of Δε*_AI_*, which was fitted separately from *K_A_* and *K_I_*), we were able to predict the input/output response of the system under a broad range of experimental conditions. For example, this framework can predict the response of the system at different repressor copy numbers *R*, repressor-operator affinities Δε*_RA_*, inducer concentrations *c*, and gene copy numbers (see Appendix A, accessible through https://doi.org/10.22002/D1.743).

### Experimental Design

We test our model by predicting the induction profiles for an array of strains that could be made using previously characterized repressor copy numbers and DNA binding energies. Our approach contrasts with previous studies that have parameterized induction curves of simple repression motifs, as these have relied on expression systems where proteins are expressed from plasmids, resulting in highly variable and unconstrained copy numbers (Murphy et al., 2007, 2010; Daber et al., 2009, 2011; Sochor, 2014). Instead, our approach relies on a foundation of previous work as depicted in Figure 1C. This includes work from our laboratory that used *E. coli* constructs based on components of the *lac* system to demonstrate how the Lac repressor (LacI) copy number *R* and operator binding energy Δε*_RA_* affect gene expression in the absence of inducer (Garcia and Phillips, 2011). Rydenfelt et al. (2014) extended the theory used in that work to the case of multiple promoters competing for a given transcription factor, which was validated experimentally by Brewster et al. (2014), who modified this system to consider expression from multiple-copy plasmids as well as the presence of competing repressor binding sites.

The present study extends this body of work by introducing three additional biophysical parameters, Δε*_AI_*, *K_A_*, and *K_I_*, which capture the allosteric nature of the transcription factor and complement the results shown by Garcia and Phillips (2011) and Brewster et al. (2014). Although the current work focuses on systems with a single site of repression, in STAR Methods, section “Inferring Allosteric Parameters from Previous Data,” we utilize data from Brewster et al. (2014) in which multiple sites of repression are explored to characterize the allosteric free energy difference Δε*_AI_* between the repressor’s active and inactive states. As explained in that section, this additional dataset is critical because multiple degenerate sets of parameters can characterize an induction curve equally well, with the Δε*_AI_* parameter compensated by the inducer dissociation constants *K_A_* and *K_I_* (see Figure S4). After fixing Δε*_AI_* as described in STAR Methods, we can use data from single-site simple repression systems to determine the values of *K_A_* and *K_I_*.

We determine the values of *K_A_* and *K_I_* by fitting to a single induction profile using Bayesian inferential methods (Sivia and Skilling, 2006). We then use Equation 5 to predict gene expression for any concentration of inducer, repressor copy number, and DNA binding energy and compare these predictions against experimental measurements. To obtain induction profiles for a set of strains with varying repressor copy numbers, we used modified *lacI* ribosomal binding sites from Garcia and Phillips (2011) to generate strains with mean repressor copy number per cell of *R* = 22 ± 4, 60 ± 20, 124 ± 30, 260 ± 40, 1,220 ± 160, and 1,740 ± 340, where the error denotes SD of at least three replicates as measured by Garcia and Phillips (2011). We note that *R* refers to the number of repressor dimers in the cell, which is twice the number of repressor tetramers reported by Garcia and Phillips (2011); since both heads of the repressor are assumed to always be either specifically or non-specifically bound to the genome, the two repressor dimers in each LacI tetramer can be considered independently. Gene expression was measured using a yellow fluorescent protein (YFP) gene, driven by a *lacUV5* promoter. Each of the six repressor copy number variants were paired with the native O1, O2, or O3 *lac* operator (Oehler et al., 1994) placed at the YFP transcription start site, thereby generating 18 unique strains. The repressor-operator binding energies (O1 Δ*ε_RA_* = −15.3 ± 0.2 *k_B_T*, O2 Δ*ε_RA_* = −13.9 ± 0.2 *k_B_T*, and O3 Δ*ε_RA_* = −9.7 ± 0.1 *k_B_T*) were previously inferred by measuring the fold-change of the *lac* system at different repressor copy numbers, where the error arises from model fitting (Garcia and Phillips, 2011). Additionally, we were able to obtain the value Δ*ε_AI_* = 4.5 *k_B_T* by fitting to previous data as discussed in STAR Methods, section “Inferring Allosteric Parameters from Previous Data”. We measure fold-change over a range of known isopropyl β-D-1-thiogalactopyranoside (IPTG) concentrations *c*, using *n* = 2 inducer binding sites per LacI dimer and approximating the number of non-specific binding sites as the length in base-pairs of the *E. coli* genome, *N_NS_* = 4.6 × 10^6^.

**Figure 3:**
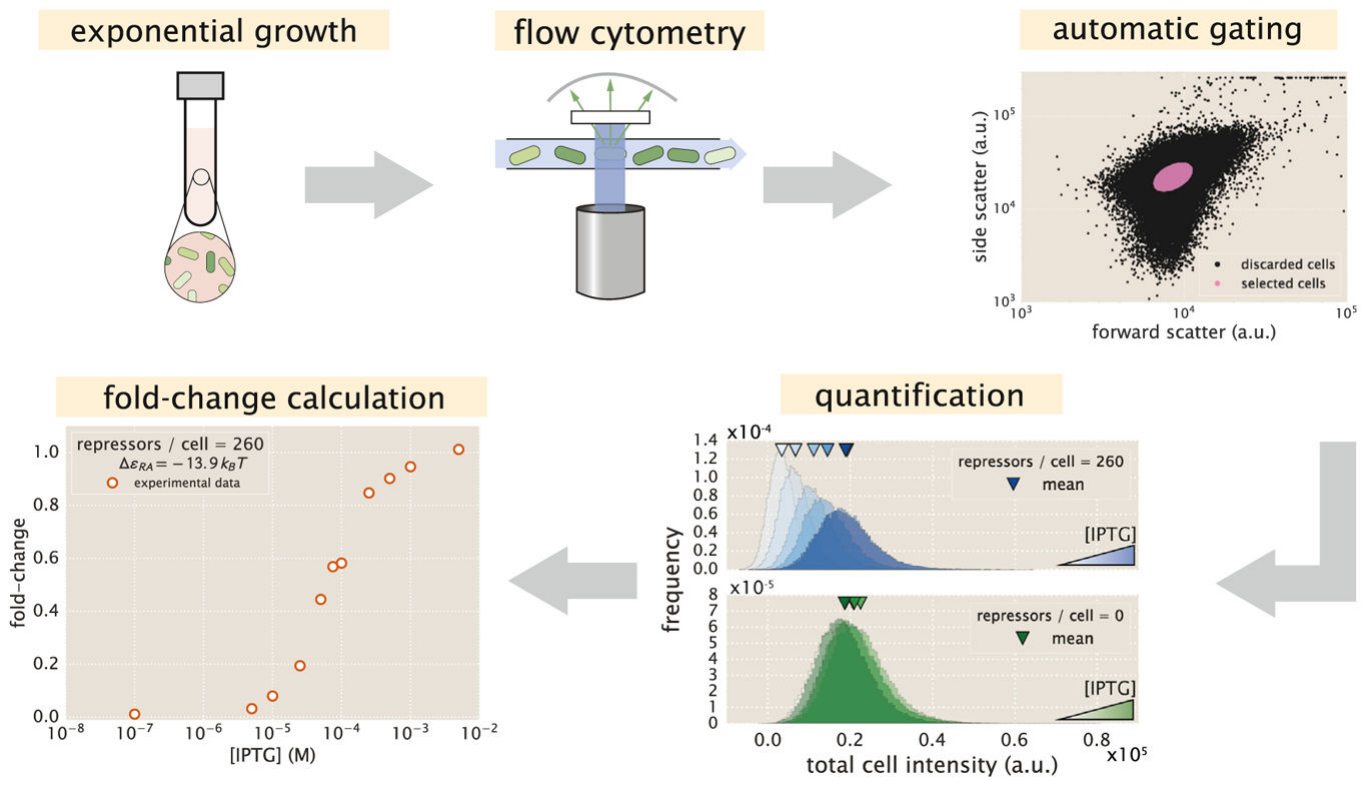
An Experimental Pipeline for High-Throughput Fold-Change Measurements Cells are grown to exponential steady state and their fluorescence is measured using flow cytometry. Automatic gating methods using forward- and side-scattering are used to ensure that all measurements come from single cells (see STAR Methods). Mean expression is then quantified at different IPTG concentrations (top, blue histograms) and for a strain without repressor (bottom, green histograms), which shows no response to IPTG as expected. Fold-change is computed by dividing the mean fluorescence in the presence of repressor by the mean fluorescence in the absence of repressor.

Our experimental pipeline for determining fold-change using flow cytometry is shown in Figure 3. In brief, cells were grown to exponential phase, in which gene expression reaches steady state (Scott et al., 2010), under concentrations of the inducer IPTG ranging between 0 and 5 mM. We measure YFP fluorescence using flow cytometry and automatically gate the data to include only single-cell measurements (see STAR Methods, section “Flow Cytometry”). To validate the use of flow cytometry, we also measured the fold-change of a subset of strains using the established method of single-cell microscopy (see Appendix B accessible through https://doi.org/10.22002/D1.743). We found that the fold-change measurements obtained from microscopy were indistinguishable from that of flow cytometry and yielded values for the inducer binding constants *K_A_* and *K_I_* that were within error.

### Determination of the *In Vivo* MWC Parameters

**Figure 4:**
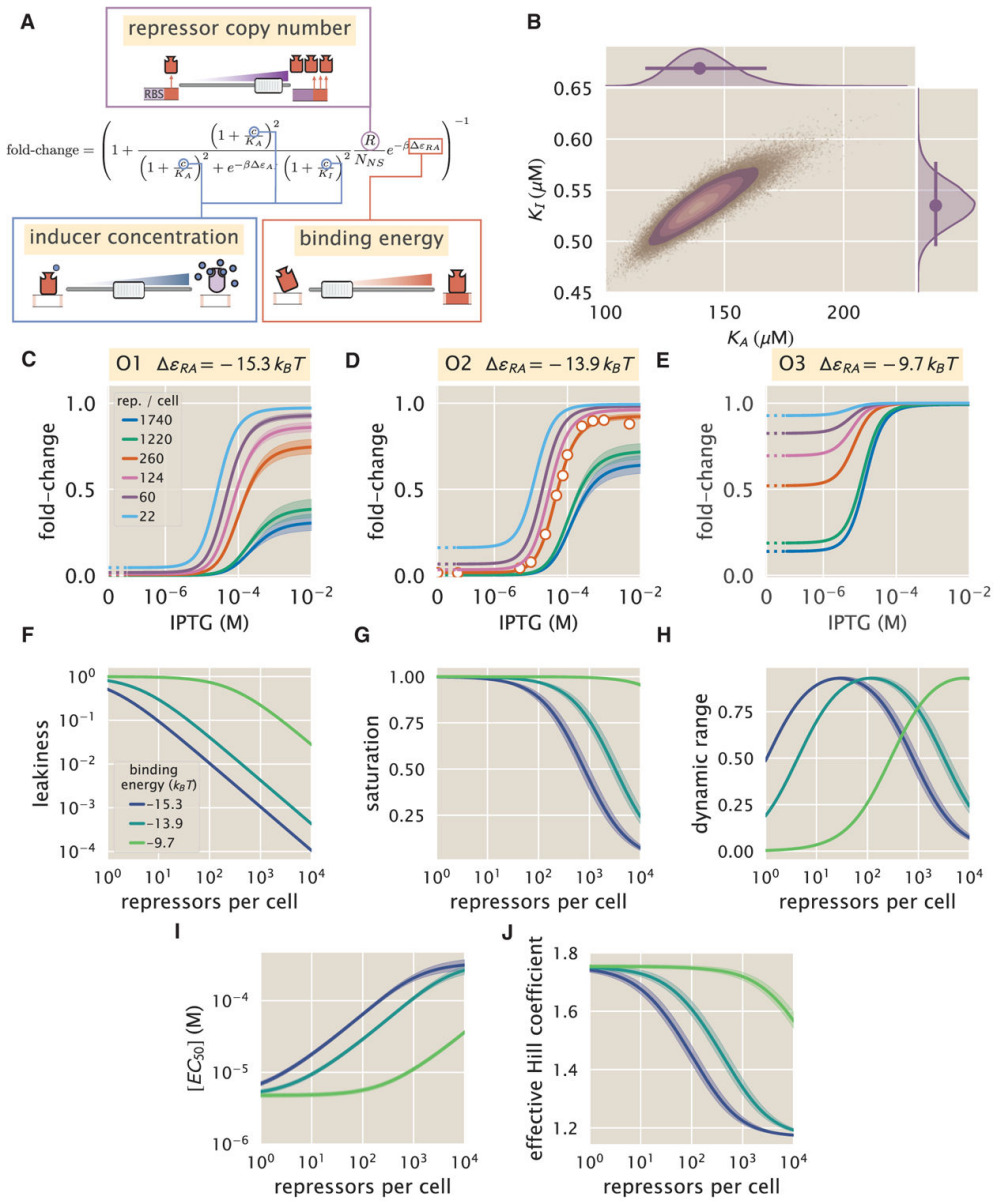
Predicting Induction Profiles for Different Biological Control Parameters (A) We can quantitatively tune *R* via ribosomal binding site (RBS) modifications, Δε*_RA_* by mutating the operator sequence, and *c* by adding different amounts of IPTG to the growth medium. (B) Previous experiments have characterized the *R*, *N_NS_*, Δε*_RA_*, and Δε*_AI_* parameters (see Figure 1C), leaving only the dissociation constants *K_A_* and *K_I_* between the inducer and the repressor in the active and inactive states, respectively, as unknown constants. These two parameters can be inferred using Bayesian parameter estimation from a single induction curve. (C–E) Predicted IPTG titration curves for different repressor copy numbers and operator strengths. Titration data for the O2 strain (white circles in D) with *R* = 260, Δ*ε_RA_* = −13.9 *k_B_T*, *n* = 2, and Δ*ε_AI_* = 4.5 *k_B_T* can be used to determine the thermodynamic parameters $K_{A}=139_{-22}^{+29}\times 10^{-6}\;M$ and $K_{I}=0.53_{-0.04}^{+0.04}\times 10^{-6}\;M$ (orange line). The remaining solid lines predict the fold-change Equation 5 for all other combinations of repressor copy numbers (shown in the legend) and repressor-DNA binding energies corresponding to the O1 operator (−15.3 *k_B_T*), O2 operator (−13.9 *k_B_T*), and O3 operator (−9.7 *k_B_T*). Error bars of experimental data show the SEM (eight or more replicates) when this error is not smaller than the diameter of the data point. The shaded regions denote the 95% credible region, although the credible region is obscured when it is thinner than the curve itself. To display the measured fold-change in the absence of inducer, we alter the scaling of the *x* axis between 0 and 10^−7^ M to linear rather than logarithmic, as indicated by a dashed line. Additionally, our model allows us to investigate key phenotypic properties of the induction profiles (see Figure 1B). (F–J) Specifically, we show predictions for the (F) leakiness, (G) saturation, (H) dynamic range, (I) [EC_50_], and (J) effective Hill coefficient of the induction profiles.

The three parameters that we tune experimentally are shown in Figure 4A, leaving the three allosteric parameters (Δε*_AI_*, *K_A_*, and *K_I_*) to be determined by fitting. We used previous LacI fold-change data (Brewster et al., 2014) to infer that Δ*ε_AI_* = 4.5 *k_B_T* (see STAR Methods, section “Inferring Allosteric Parameters from Previous Data”). Rather than fitting *K_A_* and *K_I_* to our entire dataset of 18 unique constructs, we performed Bayesian parameter estimation on data from a single strain with *R* = 260 and an O2 operator (Δ*ε_RA_* = −13.9 *k_B_T*; Garcia and Phillips, 2011) shown in Figure 4D (white circles). Using Markov chain Monte Carlo, we determine the most likely parameter values to be $K_{A}=139_{-22}^{+29}\times 10^{-6}\;M$ and $K_{I}=0.53_{-0.04}^{+0.04}\times 10^{-6}\;M$, which are the modes of their respective distributions, where the superscripts and subscripts represent the upper and lower bounds of the 95^th^ percentile of the parameter value distributions (see Figure 4B). Unfortunately, we are not able to make a meaningful value-for-value comparison of our parameters with those of earlier studies (Daber et al., 2009, 2011) because of uncertainties in both gene copy number and transcription factor copy numbers in these studies, as illustrated by the plots in Appendix A (https://doi.org/10.22002/D1.743). We then predicted the fold-change for the remaining 17 strains with no further fitting (see Figures 4C–4E) together with the specific phenotypic properties described and discussed in detail below (see Figures 4F–4J). The shaded regions in Figures 4C–4J denote the 95% credible regions. Factors determining the width of the credible regions are explored in Appendix C, accessible through https://doi.org/10.22002/D1.743.

We stress that the entire suite of predictions is based upon the induction profile of a single strain. Our ability to make such a broad range of predictions stems from the fact that our parameters of interest, such as the repressor copy number and DNA binding energy, appear as distinct physical parameters within our model. While the single dataset in Figure 4D could also be fit using a Hill function, such an analysis would be unable to predict any of the other curves in the figure (see STAR Methods, section “Alternate Characterizations of Induction”). Phenomenological expressions such as the Hill function can describe data, but lack predictive power and are thus unable to build our intuition, help us design *de novo* input-output functions, or guide future experiments (Kuhlman et al., 2007; Murphy et al., 2007).

### Comparison of Experimental Measurements with Theoretical Predictions

**Figure 5:**
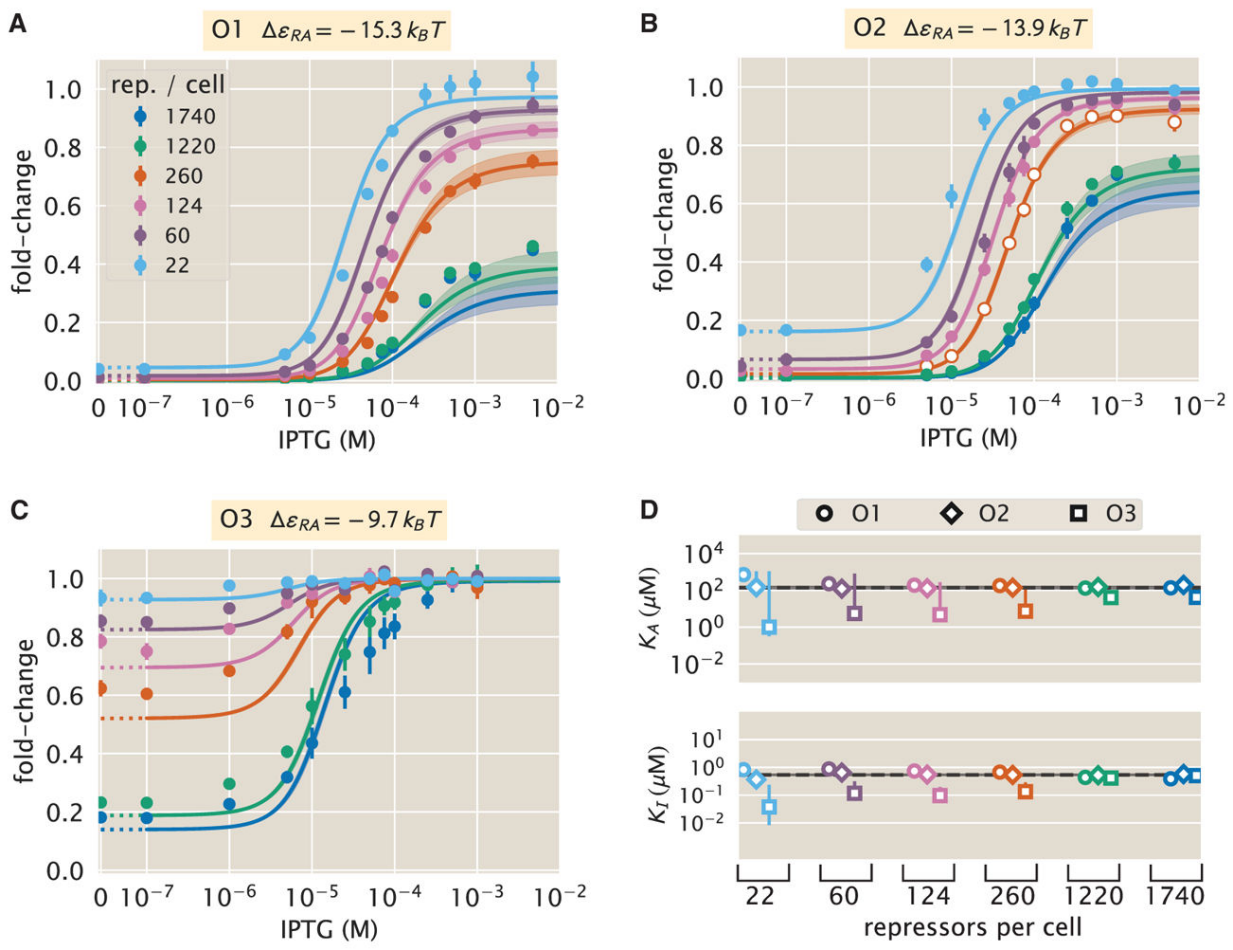
Comparison of Predictions against Measured and Inferred Data (A–C) Flow-cytometry measurements of fold-change over a range of IPTG concentrations for (A) O1, (B) O2, and (C) O3 strains at varying repressor copy numbers, overlaid on the predicted responses. Error bars for the experimental data show the SEM (eight or more replicates). As discussed in Figure 4, all of the predicted induction curves were generated prior to measurement by inferring the MWC parameters using a single dataset (the O2 strain with *R* = 260, shown by white circles in B). The predictions may therefore depend upon which strain is used to infer the parameters. (D) The inferred parameter values of the dissociation constants *K_A_* and *K_I_* using any of the 18 strains instead of the O2 strain with *R* = 260. Nearly identical parameter values are inferred from each strain, demonstrating that the same set of induction profiles would have been predicted regardless of which strain was chosen. The points show the mode, and the error bars denote the 95% credible region of the parameter value distribution. Error bars not visible are smaller than the size of the marker.

We tested the predictions shown in Figure 4 by measuring fold-change induction profiles in strains with a broad range of repressor copy numbers and repressor binding energies as characterized in Garcia and Phillips (2011). With a few notable exceptions, the results shown in Figure 5 demonstrate agreement between theory and experiment. We note that there was an apparently systematic shift in the O3 Δ*ε_RA_* = −9.7 *k_B_T* strains (Figure 5C) and all of the *R* = 1,220 and *R* = 1,740 strains. This may be partially due to imprecise previous determinations of their Δε*_RA_* and *R* values. By performing a global fit whereby we infer all parameters including the repressor copy number *R* and the binding energy Δε*_RA_*, we found better agreement for these strains, although a discrepancy in the steepness of the response for all O3 strains remains (see STAR Methods, section “Global Fit of All Parameters”). We considered a number of hypotheses to explain these discrepancies such as including other states (e.g., non-negligible binding of the inactive repressor), relaxing the weak promoter approximation, and accounting for variations in gene and repressor copy number throughout the cell cycle, but none explained the observed discrepancies. As an additional test of our model, we considered strains using the synthetic Oid operator that exhibits an especially strong binding energy of Δ*ε_RA_* = −17 *k_B_T* (Garcia and Phillips, 2011). The global fit agrees well with the Oid microscopy data, although it asserts a stronger Oid binding energy of Δ*ε_RA_* = −17.7 *k_B_T* (see Appendix D, accessible through https://doi.org/10.22002/D1.743).

To ensure that the agreement between our predictions and data is not an accident of the strain we used to perform our fitting, we also inferred *K_A_* and *K_I_* from each of the other strains. As shown in STAR Methods section “Comparison of Parameter Estimation and Fold-Change Predictions across Strains” and Figure 5D, the inferred values of *K_A_* and *K_I_* depend minimally upon which strain is chosen, indicating that these parameter values are highly robust. We also performed a global fit using the data from all 18 strains in which we fitted for the inducer dissociation constants *K_A_* and *K_I_*, the repressor copy number *R*, and the repressor-DNA binding energy Δε*_RA_* (see STAR Methods, section “Global Fit of All Parameters”). The resulting parameter values were nearly identical to those fitted from any single strain. For the remainder of the text we continue using parameters fitted from the strain with *R* = 260 repressors and an O2 operator.

### Predicting the Phenotypic Traits of the Induction Response

A subset of the properties shown in Figure 1 (i.e., the leakiness, saturation, dynamic range, [EC_50_], and effective Hill coefficient) are of significant interest to synthetic biology. For example, synthetic biology is often focused on generating large responses (i.e., a large dynamic range) or finding a strong binding partner (i.e., a small [EC_50_]) (Brophy and Voigt, 2014; Shis et al., 2014). While these properties are all individually informative, when taken together they capture the essential features of the induction response. We reiterate that a Hill function approach cannot predict these features a priori, whereas the MWC model can predict the full suite of traits as shown in Figures 4F–4J.

Using our model, Equation 5, we determine analytic expressions for the five phenotypic traits of interest. These results build upon extensive work by Martins and Swain (2011), who computed many such properties for ligand-receptor binding within the MWC model. We begin by analyzing the leakiness, which is the minimum fold-change observed in the absence of ligand, given by

(Equation 6) $$\text{leakiness}=\text{fold-change}\;(c=0)\;={\left(1+\frac{1}{1+e^{-\beta \Delta \varepsilon_{AI}}}\frac{R}{N_{NS}}e^{-\beta \Delta \varepsilon_{RA}}\right)}^{-1}$$

and the saturation, which is the maximum fold-change observed in the presence of saturating ligand,

(Equation 7) $$\text{saturation}=\text{fold-change}\;(c\to \infty )\;={\left(1+\frac{1}{1+e^{-\beta \Delta \varepsilon_{Al}}\;{\left(\frac{K_{A}}{K_{I}}\right)}^{n}}\frac{R}{N_{NS}}e^{-\beta \Delta \varepsilon_{RA}}\right)}^{-1}.$$

Systems that minimize leakiness repress strongly in the absence of effector while systems that maximize saturation have high expression in the presence of effector. Together, these two properties determine the dynamic range of a system’s response, which is given by the difference

(Equation 8) dynamicrange=saturation-leakiness.

**Figure 6:**
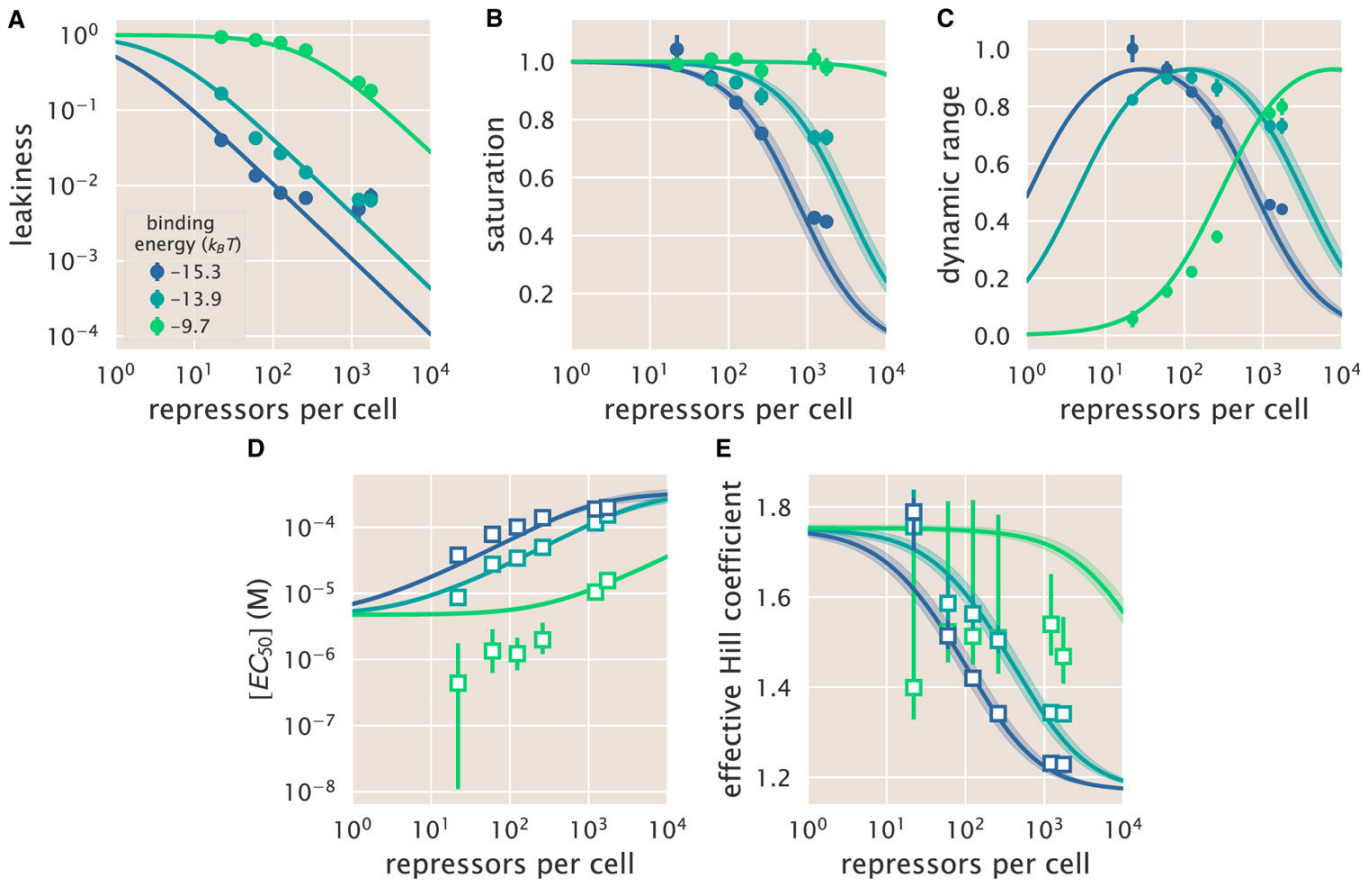
Predictions and Experimental Measurements of Key Properties of Induction Profiles (A–E) Data for the (A) leakiness, (B) saturation, and (C) dynamic range are obtained from fold-change measurements in Figure 5 in the absence of IPTG and at saturating concentrations of IPTG. The three repressor-operator binding energies in the legend correspond to the O1 operator (−15.3 *k_B_T*), O2 operator (−13.9 *k_B_T*), and O3 operator (−9.7 *k_B_T*). Both the (D) [EC_50_] and (E) effective Hill coefficient are inferred by individually fitting each operator-repressor pairing in Figures 5A–5C separately to Equation 5 in order to smoothly interpolate between the data points. Error bars in (A) to (C) represent the SEM for eight or more replicates; error bars in (D) and (E) represent the 95% credible region for the parameter found by propagating the credible region of our estimates of *K_A_* and *K_I_* into Equations 9 and 10.

These three properties are shown in Figures 4F–4H. We discuss these properties in greater detail in STAR Methods, section “Properties of Induction Titration Curves.” Figures 6A–6C show that the measurements of these three properties, derived from the fold-change data in the absence of IPTG and the presence of saturating IPTG, closely match the predictions for all three operators.

Two additional properties of induction profiles are the [EC_50_] and effective Hill coefficient, which determine the range of inducer concentration in which the system’s output goes from its minimum to maximum value. The [EC_50_] denotes the inducer concentration required to generate a system response Equation 5 halfway between its minimum and maximum value,

(Equation 9) $$\text{fold-change}\;(c=[{\text{EC}}_{50}])=\frac{\text{leakiness}+\text{saturation}}{2}.$$

The effective Hill coefficient *h*, which quantifies the steepness of the curve at the [EC_50_] (Marzen et al., 2013), is given by

(Equation 10) $$h={\left(2\frac{d}{d\text{log}(c)}\;\left[\text{log}\left(\frac{\text{fold-change}\;(c)-\text{leakiness}}{\text{dynamic}\;\text{range}}\right)\right]\right)}_{c=[{\text{EC}}_{50}]}.$$

Figures 4I and 4J shows how the [EC_50_] and effective Hill coefficient depend on the repressor copy number. In STAR Methods section “Properties of Induction Titration Curves,” we discuss the analytic forms of these two properties as well as their dependence on the repressor-DNA binding energy.

Figures 6D and 6E shows the estimated values of the [EC_50_] and the effective Hill coefficient overlaid on the theoretical predictions. Both properties were obtained by fitting Equation 5 to each individual titration curve and computing the [EC_50_] and effective Hill coefficient using Equations 9 and 10, respectively. We find that the predictions made with the single strain fit closely match those made for each of the strains with O1 and O2 operators, but the predictions for the O3 operator are markedly off. In STAR Methods section “Alternate Characterizations of Induction,” we show that the large, asymmetric error bars for the O3 *R* = 22 strain arise from its nearly flat response, where the lack of dynamic range makes it impossible to determine the value of the inducer dissociation constants *K_A_* and *K_I_*, as can be seen in the uncertainty of both the [EC_50_] and effective Hill coefficient. Discrepancies between theory and data for O3 are improved, but not fully resolved, by performing a global fit or fitting the MWC model individually to each curve (see STAR Methods, sections “Global Fit of All Parameters” and “Comparison of Parameter Estimation and Fold-Change Predictions across Strains”). It remains an open question as to how to account for discrepancies in O3, in particular regarding the significant mismatch between the predicted and fitted effective Hill coefficients.

### Data Collapse of Induction Profiles

Our primary interest heretofore was to determine the system response at a specific inducer concentration, repressor copy number, and repressor-DNA binding energy. However, the cell does not necessarily “care about” the precise number of repressors in the system or the binding energy of an individual operator. The relevant quantity for cellular function is the fold-change enacted by the regulatory system. This raises the question: given a specific value of the fold-change, what combination of parameters will give rise to this desired response? In other words, what trade-offs between the parameters of the system will produce the same mean cellular output? These are key questions both for understanding how the system is governed and for engineering specific responses in a synthetic biology context. To address these questions, we follow the data collapse strategy used in a number of previous studies (Sourjik and Berg, 2002; Keymer et al., 2006; Swem et al., 2008), and rewrite Equation 5 as a Fermi function,

(Equation 11) $$\text{fold-change}=\frac{1}{1+e^{-F(c)}},$$

where *F*(*c*) is the free energy of the repressor binding to the operator of interest relative to the unbound operator state in *k_B_T* units (Keymer et al., 2006; Swem et al., 2008; Phillips, 2015), which is given by

(Equation 12) $$F(c)=\frac{\Delta \varepsilon_{RA}}{k_{B}T}-\log\frac{{\left(1+\frac{c}{K_{A}}\right)}^{n}}{{\left(1+\frac{c}{K_{A}}\right)}^{n}+e^{-\beta \Delta \varepsilon_{AI}}\;{\left(1+\frac{c}{K_{I}}\right)}^{n}}-\log\frac{R}{N_{NS}}.$$

The first term in *F*(*c*) denotes the repressor-operator binding energy, the second the contribution from the inducer concentration, and the last the effect of the repressor copy number. We note that elsewhere, this free energy has been dubbed the Bohr parameter since such families of curves are analogous to the shifts in hemoglobin binding curves at different pHs known as the Bohr effect (Mirny, 2010; Phillips, 2015; Einav et al., 2016).

**Figure 7:**
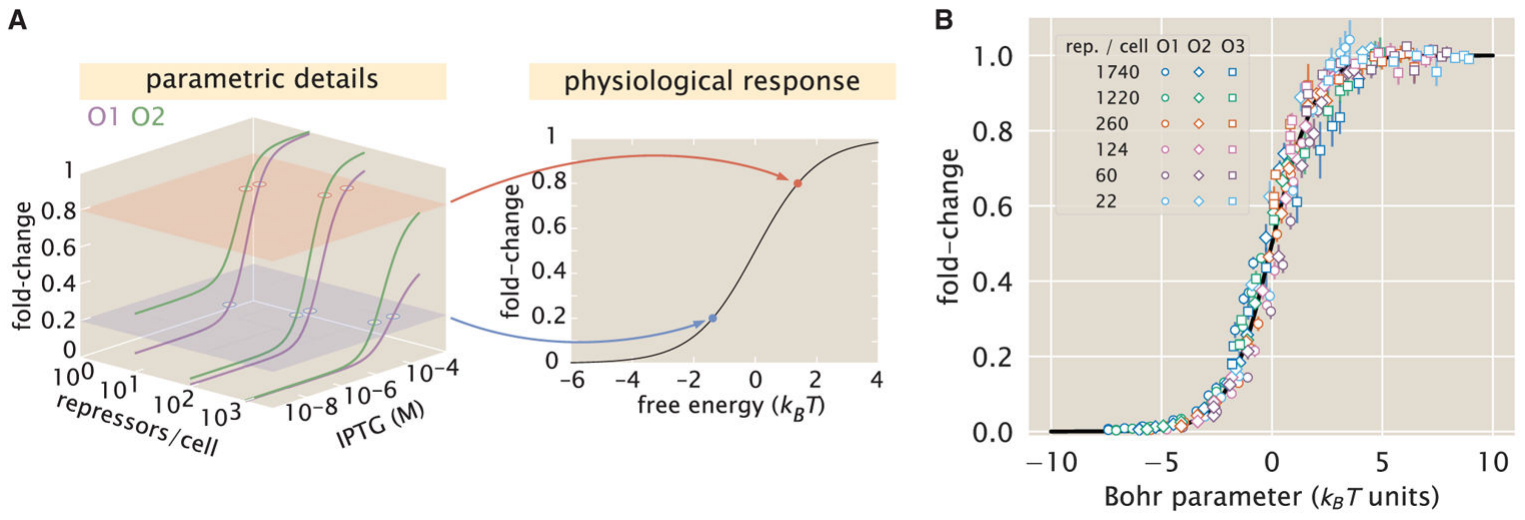
Fold-Change Data from a Broad Collection of Different Strains Collapse onto a Single Master Curve (A) Any combination of parameters can be mapped to a single physiological response (i.e., fold-change) via the free energy, which encompasses the parametric details of the model. (B) Experimental data from Figure 5 collapse onto a single master curve as a function of the free energy Equation 12. The free energy for each strain was calculated from Equation 12 using *n* = 2, Δ*ε_AI_* = 4.5 *k_B_T*, *K_A_* = 139 × 10^−6^ M, *K_I_* = 0.53 × 10^−6^ M, and the strain-specific *R* and Δε*_RA_*. All data points represent the mean, and error bars are the SEM for eight or more replicates.

Instead of analyzing each induction curve individually, the free energy provides a natural means to simultaneously characterize the diversity in our 18 induction profiles. Figure 7A demonstrates how the various induction curves from Figures 4C–4E all collapse onto a single master curve, where points from every induction profile that yield the same fold-change are mapped onto the same free energy. Figure 7B shows this data collapse for the 216 data points in Figures 5A–5C, demonstrating the close match between the theoretical predictions and experimental measurements across all 18 strains.

There are many different combinations of parameter values that can result in the same free energy as defined in Equation 12. For example, suppose a system originally has a fold-change of 0.2 at a specific inducer concentration and then operator mutations increase the Δε*_RA_* binding energy (Garcia et al., 2012). While this serves to initially increase both the free energy and the fold-change, a subsequent increase in the repressor copy number could bring the cell back to the original fold-change level. Such trade-offs hint that there need not be a single set of parameters that evoke a specific cellular response, but rather that the cell explores a large but degenerate space of parameters with multiple, equally valid paths.

## DISCUSSION

Since the early work by Monod, Wyman, and Changeux (Monod et al., 1963, 1965), an array of biological phenomena have been tied to the existence of macromolecules that switch between inactive and active states. Examples can be found in a wide variety of cellular processes, including ligand-gated ion channels (Auerbach, 2012), enzymatic reactions (Velyvis et al., 2007; Einav et al., 2016), chemotaxis (Keymer et al., 2006), quorum sensing (Swem et al., 2008), G-protein-coupled receptors (Canals et al., 2012), physiologically important proteins (Milo et al., 2007; Levantino et al., 2012), and beyond. One of the most ubiquitous examples of allostery is in the context of gene expression, where an array of molecular players bind to transcription factors to influence their ability to regulate gene activity (Huang et al., 2011; Li et al., 2014). A number of studies have focused on developing a quantitative understanding of allosteric regulatory systems. Martins and Swain (2011) and Marzen et al. (2013) analytically derived fundamental properties of the MWC model, including the leakiness and dynamic range described in this work, noting the inherent trade-offs in these properties when tuning the model’s parameters. Work in the Church and Voigt labs, among others, has expanded on the availability of allosteric circuits for synthetic biology (Lutz and Bujard, 1997; Moon et al., 2012; Rogers et al., 2015; Rohlhill et al., 2017). Recently, Daber et al. (2009) theoretically explored the induction of simple repression within the MWC model and experimentally measured how mutations alter the induction profiles of transcription factors (Daber et al., 2011). Vilar and Saiz analyzed a variety of interactions in inducible *lac*-based systems including the effects of oligomerization and DNA folding on transcription factor induction (Saiz and Vilar, 2008; Vilar and Saiz, 2013). Other work has attempted to use the *lac* system to reconcile *in vitro* and *in vivo* measurements (Tungtur et al., 2011; Sochor, 2014).

Although this body of work has done much to improve our understanding of allosteric transcription factors, there have been few attempts to explicitly connect quantitative models to experiments. Here, we generate a predictive model of allosteric transcriptional regulation and then test the model against a thorough set of experiments using well-characterized regulatory components. Specifically, we used the MWC model to build upon a well-established thermodynamic model of transcriptional regulation (Bintu et al., 2005a; Garcia and Phillips, 2011), allowing us to compose the model from a minimal set of biologically meaningful and experimentally accessible parameters. We argue that one would not be able to generate such a wide array of quantitative predictions by using a Hill function, which abstracts away the biophysical meaning of the parameters into phenomenological parameters (Forsén and Linse, 1995). Furthermore, our model reveals systematic relationships between behaviors that previously were only determined empirically.

One such property is the dynamic range, which is of considerable interest when designing or characterizing a genetic circuit, and is revealed to have an interesting property: although changing the value of Δε*_RA_* causes the dynamic range curves to shift to the right or left, each curve has the same shape and in particular the same maximum value. This means that strains with strong or weak binding energies can attain the same dynamic range when the value of *R* is tuned to compensate for the binding energy. This feature is not immediately apparent from the IPTG induction curves, which show very low dynamic ranges for several of the O1 and O3 strains. Without the benefit of models that can predict such phenotypic traits, efforts to engineer genetic circuits with allosteric transcription factors must rely on trial and error to achieve specific responses (Rogers et al., 2015; Rohlhill et al., 2017). Other calculable properties, such as leakiness, saturation, [EC_50_], and the effective Hill coefficient, agree well with experimental measurement. One exception is the titration profile of the weakest operator, O3. While performing a global fit for all model parameters marginally improves the prediction of all properties for O3 (see STAR Methods, section “Global Fit of All Parameters”), a noticeable difference remains when inferring the effective Hill coefficient or the [EC_50_]. We further tried including additional states (such as allowing the inactive repressor to bind to the operator), relaxing the weak promoter approximation, accounting for changes in gene and repressor copy number throughout the cell cycle (Jones et al., 2014), and refitting the original binding energies from Garcia et al. (2011), but such generalizations were unable to account for the O3 data. It remains an open question as to how the discrepancy between the theory and measurements for O3 can be reconciled.

Despite the diversity observed in the induction profiles of each of our strains, our data are unified by their reliance on fundamental biophysical parameters. In particular, we have shown that our model for fold-change can be rewritten in terms of the free energy Equation 12, which encompasses all of the physical parameters of the system. This has proved to be an illuminating technique in a number of studies of allosteric proteins (Sourjik and Berg, 2002; Keymer et al., 2006; Swem et al., 2008). Although it is experimentally straightforward to observe system responses to changes in effector concentration *c*, framing the input-output function in terms of *c* can give the misleading impression that changes in system parameters lead to fundamentally altered system responses. Alternatively, if one can find the “natural variable” that enables the output to collapse onto a single curve, it becomes clear that the system’s output is not governed by individual system parameters, but rather the contributions of multiple parameters that define the natural variable. Plotting the fold-change data against their respective free energies leads to a clean collapse onto a single curve (see Figure 7). This enables us to analyze how parameters can compensate each other. For example, rather than viewing strong repression as a consequence of low IPTG concentration *c* or high repressor copy number *R*, we can now observe that strong repression is achieved when the free energy *F*(*c*) ≤ −5 *k_B_T*, a condition which can be reached in a number of ways.

While our experiments validated the theoretical predictions in the case of simple repression, we expect the framework presented here to apply much more generally to different biological instances of allosteric regulation. For example, we can use this model to study more complex systems such as when transcription factors interact with multiple operators (Bintu et al., 2005a). We can further explore different regulatory configurations such as corepression, activation, and coactivation, each of which are found in *E. coli* (see Appendix E, accessible through https://doi.org/10.22002/D1.743). This work can also serve as a springboard to characterize not just the mean but the full gene expression distribution and thus quantify the impact of noise on the system (Eldar and Elowitz, 2010). Another extension of this approach would be to theoretically predict and experimentally verify whether the repressor-inducer dissociation constants *K_A_* and *K_I_* or the energy difference Δε*_AI_* between the allosteric states can be tuned by making single amino acid substitutions in the transcription factor (Daber et al., 2011; Phillips, 2015). Finally, we expect that the kind of rigorous quantitative description of the allosteric phenomenon provided here will make it possible to construct biophysical models of fitness for allosteric proteins similar to those already invoked to explore the fitness effects of transcription factor binding site strengths and protein stability (Gerland and Hwa, 2002; Berg et al., 2004; Zeldovich and Shakhnovich, 2008). In total, our approach shows that a thermodynamic formulation of the MWC model supersedes phenomenological fitting functions for understanding transcriptional regulation by allosteric proteins.

## STAR★METHODS

### KEY RESOURCES TABLE

**Table:** 

REAGENT or RESOURCE	SOURCE	IDENTIFIER
Software and Algorithms		
	GitHub Repository	DOI 10.5281/zenodo.1163620
Additional Supplemental Information	CaltechDATA Repository	https://doi.org/10.22002/D1.743

### CONTACT FOR REAGENT AND RESOURCE SHARING

Further information and requests for resources and reagents should be directed to and will be fulfilled by the Lead Contact, Rob Phillips (phillips@pboc.caltech.edu).

### EXPERIMENTAL MODEL AND SUBJECT DETAILS

#### Bacterial Strains and DNA Constructs

All strains used in these experiments were derived from *E. coli* K12 MG1655 with the *lac* operon removed, adapted from those created and described in Garcia and Phillips (2011). Briefly, the operator variants and YFP reporter gene were cloned into a pZS25 background which contains a *lacUV5* promoter that drives expression as is shown schematically in Figure 2. These constructs carried a kanamycin resistance gene and were integrated into the *galK* locus of the chromosome using *λ* Red recombineering (Sharan et al., 2009). The *lacI* gene was constitutively expressed via a P_LtetO-1_ promoter (Lutz and Bujard, 1997), with ribosomal binding site mutations made to vary the LacI copy number as described in Salis et al. (2009) using site-directed mutagenesis (Quickchange II; Stratagene), with further details in Garcia and Phillips (2011). These *lacI* constructs carried a chloramphenicol resistance gene and were integrated into the *ybcN* locus of the chromosome. Final strain construction was achieved by performing repeated P1 transduction (Thomason et al., 2007) of the different operator and *lacI* constructs to generate each combination used in this work. Integration was confirmed by PCR amplification of the replaced chromosomal region and by sequencing. Primers and final strain genotypes are listed in Tables S1 and S2, respectively.

It is important to note that the rest of the *lac* operon (*lacZYA*) was never expressed. The LacY protein is a transmembrane protein which actively transports lactose as well as IPTG into the cell. As LacY was never produced in our strains, we assume that the extracellular and intracellular IPTG concentration was approximately equal due to diffusion across the membrane into the cell as is suggested by previous work (Fernández-Castané et al., 2012).

To make this theory applicable to transcription factors with any number of DNA binding domains, we used a different definition for repressor copy number than has been used previously. We define the LacI copy number as the average number of repressor dimers per cell whereas in Garcia and Phillips (2011), the copy number is defined as the average number of repressor tetramers in each cell. To motivate this decision, we consider the fact that the LacI repressor molecule exists as a tetramer in *E. coli* (Lewis et al., 1996) in which a single DNA binding domain is formed from dimerization of LacI proteins, so that wild-type LacI might be described as dimer of dimers. Since each dimer is allosterically independent (i.e., either dimer can be allosterically active or inactive, independent of the configuration of the other dimer) (Daber et al., 2009), a single LacI tetramer can be treated as two functional repressors. Therefore, we have simply multiplied the number of repressors reported in Garcia and Phillips (2011) by a factor of two. This factor is included as a keyword argument in the numerous Python functions used to perform this analysis, as discussed in the code documentation.

A subset of strains in these experiments were measured using fluorescence microscopy for validation of the flow cytometry data and results. To aid in the high-fidelity segmentation of individual cells, the strains were modified to constitutively express an mCherry fluorophore. This reporter was cloned into a pZS4*1 backbone (Lutz and Bujard, 1997) in which mCherry is driven by the *lacUV5* promoter. All microscopy and flow cytometry experiments were performed using these strains.

#### Growth Conditions for Flow Cytometry Measurements

All measurements were performed with *E. coli* cells grown to mid-exponential phase in standard M9 minimal media (M9 5X Salts, Sigma-Aldrich M6030; 2 mM magnesium sulfate, Mallinckrodt Chemicals 6066-04; 100 *μ*M calcium chloride, Fisher Chemicals C79-500) supplemented with 0.5% (w/v) glucose. Briefly, 500 *μ*L cultures of *E. coli* were inoculated into Lysogeny Broth (LB Miller Powder, BD Medical) from a 50% glycerol frozen stock (−80°C) and were grown overnight in a 2 mL 96-deep-well plate sealed with a breathable nylon cover (Lab Pak - Nitex Nylon, Sefar America, Cat. No. 241205) with rapid agitation for proper aeration. After approximately 12 to 15 hr, the cultures had reached saturation and were diluted 1000-fold into a second 2 mL 96-deep-well plate where each well contained 500 *μ*L of M9 minimal media supplemented with 0.5% w/v glucose (anhydrous D-Glucose, Macron Chemicals) and the appropriate concentration of IPTG (Isopropyl *β*-D-1-thiogalactopyranoside, Dioxane Free, Research Products International). These were sealed with a breathable cover and were allowed to grow for approximately 8 hr. Cells were then diluted ten-fold into a round-bottom 96-well plate (Corning Cat. No. 3365) containing 90 *μ*L of M9 minimal media supplemented with 0.5% w/v glucose along with the corresponding IPTG concentrations. For each IPTG concentration, a stock of 100-fold concentrated IPTG in double distilled water was prepared and partitioned into 100 *μ*L aliquots. The same parent stock was used for all experiments described in this work.

#### *E. coli* Primer and Strain List

Here we provide additional details about the genotypes of the strains used, as well as the primer sequences used to generate them. *E. coli* strains were derived from K12 MG1655. For those containing *R* = 22, we used strain HG104 which additionally has the *lacYZA* operon deleted (positions 360,483 to 365,579) but still contains the native *lacI* locus. All other strains used strain HG105, where both the *lacYZA* and *lacI* operons have both been deleted (positions 360,483 to 366,637).

All 25x+11-yfp expression constructs were integrated at the *galK* locus (between positions 1,504,078 and 1,505,112) while the 3*1x-lacI constructs were integrated at the *ybcN* locus (between positions 1,287,628 and 1,288,047). Integration was performed with *λ* Red recombineering (Sharan et al., 2009) as described in Garcia and Phillips (2011) using the primers listed in Table S1. We follow the notation of Lutz and Bujard (Lutz and Bujard, 1997) for the nomenclature of the different constructs used. Specifically, the first number refers to the antibiotic resistance cassette that is present for selection (2 = kanamycin, 3 = chloramphenicol, and 4 = spectinomycin) and the second number refers to the promoter used to drive expression of either YFP or LacI (1 = P_LtetO-1_, and 5 = *lacUV5*). Note that in 25x+11-yfp, x refers to the LacI operator used, which is centered at +11 (or alternatively, begins at the transcription start site). For the different LacI constructs, 3*1x-lacI, x refers to the different ribosomal binding site modifications that provide different repressor copy numbers and follows from Garcia and Phillips (2011). The asterisk refers to the presence of FLP recombinase sites flanking the chloramphenicol resistance gene that can be used to lose this resistance. However, we maintained the resistance gene in our constructs. A summary of the final genotypes of each strain is listed in Table S2. In addition, each strain also contained the plasmid pZS4*1-mCherry and provided constitutive expression of the mCherry fluorescent protein. This pZS plasmid is a low copy (SC101 origin of replication) where like with 3*1x-lacI, mCherry is driven by a P_LtetO-1_ promoter.

### METHOD DETAILS

In this method details section we provide extensive and rigorous explanation of both the theoretical and experimental results shown in this work. First in the “Flow Cytometry” section we detail the specifications of the equipment and the corresponding settings used to experimentally determine the fold-change in gene expression. We also provide an explanation of the pipeline used to process the raw data, and compare the flow cytometry results with other indirect measurements of gene expression.

In the next section “Inferring Allosteric Parameters from Previous Data” we specify how we inferred the free energy difference between the active and inactive state of the repressor using data from Brewster et al. (2014). In combination with an extension of the theory that accounts for competition for transcription factors between multiple binding sites we show how this data can lead to an estimate of the Δε*_AI_* parameter from the model.

The “Alternate Characterizations of Induction” section explores the use of alternative formulations for the allosteric nature of the transcriptional repressor. By comparing our MWC formulation with the Hill function we explain the advantages and limitations of the approach presented in the main text.

For the “Global Fit of All Parameters” section we follow a different procedure than the one followed in the main text in which only two parameters were fit to a single data set. In this section we use all of the experimental data and perform a Bayesian parameter inference where all model parameters including the repressor copy number and the repressor-DNA binding energy are allowed to vary. By doing so we show that the minimum set of parameters fit in the main text gives almost as good characterization as including all the extra degrees of freedom.

In section “Comparison of Parameter Estimation and Fold-Change Predictions across Strains” we perform a cross-comparison of the fitting procedure followed in the main text in which we use each of the single strains to fit the dissociation constants of the inducer, *K_A_* and *K_I_*, and use these values to predict the rest of the strains with the same operator. This comparison aims to show how the characterization of these dissociation constants is for the most part independent of the strain chosen for the fit as long as there is enough dynamic range in the strain to get a reliable estimate of these parameters.

Finally, in section “Properties of Induction Titration Curves” we derive the theoretical expressions for the induction curve properties shown in Figures 4 and 6.

#### Flow Cytometry

In this section, we provide information regarding the equipment used to make experimental measurements of the fold-change in gene expression in the interests of transparency and reproducibility. We also provide a summary of our unsupervised method of gating the flow cytometry measurements for consistency between experimental runs.

##### Equipment

Due to past experience using the Miltenyi Biotec MACSQuant flow cytometer during the Physiology summer course at the Marine Biological Laboratory, we used the same flow cytometer for the formal measurements in this work graciously provided by the Pamela Björkman lab at Caltech. All measurements were made using an excitation wavelength of 488 nm with an emission filter set of 525/50 nm. This excitation wavelength provides approximately 40% of the maximum YFP absorbance (Chroma Technology Corporation, 2016), and this was found to be sufficient for the purposes of these experiments. A useful feature of modern flow cytometry is the high-sensitivity signal detection through the use of photomultiplier tubes (PMT) whose response can be tuned by adjusting the voltage. Thus, the voltage for the forward-scatter (FSC), side-scatter (SSC), and gene expression measurements were tuned manually to maximize the dynamic range between autofluorescence signal and maximal expression without losing the details of the population distribution. Once these voltages were determined, they were used for all subsequent measurements. Extremely low signal producing particles were discarded before data storage by setting a basal voltage threshold, thus removing the majority of spurious events. The various instrument settings for data collection are given in Table S3.

##### Experimental Measurement

Prior to each day’s experiments, the analyzer was calibrated using MACSQuant Calibration Beads (Cat. No. 130-093-607) such that day-to-day experiments would be comparable. A single data set consisted of seven bacterial strains, all sharing the same operator, with varying repressor copy numbers (*R* = 0, 22, 60, 124, 260, 1220, and 1740), in addition to an autofluorescent strain, under twelve IPTG concentrations. Data collection took place over 2 to 3 hr. During this time, the cultures were held at approximately 4°C by placing the 96-well plate on a MACSQuant ice block. Because the ice block thawed over the course of the experiment, the samples measured last were approximately at room temperature. This means that samples may have grown slightly by the end of the experiment. To confirm that this continued growth did not alter the measured results, a subset of experiments were run in reverse meaning that the fully induced cultures were measured first and the uninduced samples last. The plate arrangements and corresponding fold-change measurements are shown in Figures S1A and S1B, respectively. The measured fold-change values in the reverse ordered plate appear to be drawn from the same distribution as those measured in the forward order, meaning that any growth that might have taken place during the experiment did not significantly affect the results. Both the forward and reverse data sets were used in our analysis.

##### Unsupervised Gating

Flow cytometry data will frequently include a number of spurious events or other undesirable data points such as cell doublets and debris. The process of restricting the collected data set to those data determined to be “real” is commonly referred to as gating. These gates are typically drawn manually (Maecker et al., 2005) and restrict the data set to those points which display a high degree of linear correlation between their forward-scatter (FSC) and side-scatter (SSC). The development of unbiased and unsupervised methods of drawing these gates is an active area of research (Lo et al., 2008; Aghaeepour et al., 2013).

For this study, we used an automatic unsupervised gating procedure to filter the flow cytometry data based on the front and side-scattering values returned by the MACSQuant flow cytometer. We assume that the region with highest density of points in these two channels corresponds to single-cell measurements. Everything extending outside of this region was discarded in order to exclude sources of error such as cell clustering, particulates, or other spurious events.

In order to define the gated region we fit a two-dimensional Gaussian function to the log_10_ forward-scattering (FSC) and the log_10_ side-scattering (SSC) data. We then kept a fraction *α* ∈ [0, 1] of the data by defining an elliptical region given by

(Equation 13) $${\left(x-\mu \right)}^{T}\sum^{-1}(x-\mu )\leq \chi_{\alpha }^{2}(p),$$

where **x** is the 2 × 1 vector containing the log(FSC) and log(SSC), **μ** is the 2 × 1 vector representing the mean values of log(FSC) and log(SSC) as obtained from fitting a two-dimensional Gaussian to the data, and **Σ** is the 2 × 2 covariance matrix also obtained from the Gaussian fit. $\chi_{\alpha }^{2}(p)$ is the quantile function for probability *p* of the chi-squared distribution with two degrees of freedom. Figure S2 shows an example of different gating contours that would arise from different values of *α* in Equation 13. In this work, we chose *α* = 0.4 which we deemed was a sufficient constraint to minimize the noise in the data. As explained in Appendix B on https://doi.org/10.22002/D1.743 in we compared our high throughput flow cytometry data with single cell microscopy, confirming that the automatic gating did not introduce systematic biases to the analysis pipeline. The specific code where this gating is implemented can be found in GitHub repository (http://doi.org/10.5281/zenodo.1163620).

##### Comparison of Flow Cytometry with Other Methods

Previous work from our lab experimentally determined fold-change for similar simple repression constructs using a variety of different measurement methods (Garcia et al., 2011; Brewster et al., 2014). Garcia and Phillips used the same background strains as the ones used in this work, but gene expression was measured with Miller assays based on colorimetric enzymatic reactions with the LacZ protein (Garcia and Phillips, 2011). Brewster et al. (2014) used a LacI dimer with the tetramerization region replaced with an mCherry tag, where the fold-change was measured as the ratio of the gene expression rate rather than a single snapshot of the gene output.

Figure S3 shows the comparison of these methods along with the flow cytometry method used in this work. The consistency of these three readouts validates the quantitative use of flow cytometry and unsupervised gating to determine the fold-change in gene expression. However, one important caveat revealed by this figure is that the sensitivity of flow cytometer measurements is not sufficient to accurately determine the fold-change for the high repressor copy number strains in O1 without induction. Instead, a method with a large dynamic range such as the Miller assay is needed to accurately resolve the fold-change at such low expression levels.

#### Inferring Allosteric Parameters from Previous Data

The fold-change profile described by Equation 5 features three unknown parameters *K_A_*, *K_I_*, and Δε*_AI_*. In this section, we explore different conceptual approaches to determining these parameters. We first discuss how the induction titration profile of the simple repression constructs used in this paper are not sufficient to determine all three MWC parameters simultaneously, since multiple degenerate sets of parameters can produce the same fold-change response. We then utilize an additional data set from Brewster et al. (2014) to determine the parameter Δ*ε_AI_* = 4.5 *k_B_T*, after which the remaining parameters *K_A_* and *K_I_* can be extracted from any induction profile with no further degeneracy.

##### Degenerate Parameter Values

In this section, we discuss how multiple sets of parameters may yield identical fold-change profiles. More precisely, we shall show that if we try to fit the data in Figure 4C to the fold-change Equation 5 and extract the three unknown parameters (*K_A_*, *K_I_*, and Δε*_AI_*), then multiple degenerate parameter sets would yield equally good fits. In other words, this data set alone is insufficient to uniquely determine the actual physical parameter values of the system. This problem persists even when fitting multiple data sets simultaneously as in Section “Global Fit of All Parameters”.

In Figure S4A, we fit the *R* = 260 data by fixing Δε*_AI_* to the value shown on the *x*-axis and determine the parameters *K_A_* and *K_I_* given this constraint. We use the fold-change function Equation 5 but with *β*Δε*_RA_* modified to the form *β*Δ*ε̃_RA_* in Equation 5 to account for the underlying assumptions used when fitting previous data (see Section “Computing Δε*_AI_*” for a full explanation of why this modification is needed).

The best-fit curves for several different values of Δε*_AI_* are shown in Figure S4B. Note that these fold-change curves are nearly overlapping, demonstrating that different sets of parameters can yield nearly equivalent responses. Without more data, the relationships between the parameter values shown in Figure S4A represent the maximum information about the parameter values that can be extracted from the data. Additional experiments, which independently measure any of these unknown parameters, could resolve this degeneracy. For example, NMR measurements could be used to directly measure the fraction (1 + *e*^−*β*Δ*ε*_*AI*_^)^−1^ of active repressors in the absence of IPTG (Gardino et al., 2003; Boulton and Melacini, 2016).

##### Computing Δε*_AI_*

As shown in the previous section, the fold-change response of a single strain is not sufficient to determine the three MWC parameters (*K_A_*, *K_I_*, and Δε*_AI_*), since degenerate sets of parameters yield nearly identical fold-change responses. To circumvent this degeneracy, we now turn to some previous data from the *lac* system in order to determine the value of Δε*_AI_*. Specifically, we consider two previous sets of work from: (1) Garcia and Phillips (2011) and (2) Brewster et al. (2014), both of which measured fold-change with the same simple repression system in the absence of inducer (*c* = 0) but at various repressor copy numbers *R*. The original analysis for both data sets assumed that in the absence of inducer all of the Lac repressors were in the active state. As a result, the effective binding energies they extracted were a convolution of the DNA binding energy Δε*_RA_* and the allosteric energy difference Δε*_AI_* between the Lac repressor’s active and inactive states. We refer to this convoluted energy value as Δ*ε̃_RA_*. We first disentangle the relationship between these parameters in Garcia and Phillips and then use this relationship to extract the value of Δε*_AI_* from the Brewster et al. dataset.

Garcia and Phillips determined the total repressor copy numbers *R* of different strains using quantitative western blots. Then they measured the fold-change at these repressor copy numbers for simple repression constructs carrying the O1, O2, O3, and Oid *lac* operators integrated into the chromosome. These data were then fit to the following thermodynamic model to determine the repressor-DNA binding energies Δ*ε̃_RA_* for each operator,

(Equation 14) $$\text{fold-change}(c=0)={\left(1+\frac{R}{N_{NS}}e^{-\beta \Delta {\tilde{\varepsilon }}_{RA}}\right)}^{-1}.$$

Note that this functional form does not exactly match our fold-change Equation 5 in the limit *c*=0,

(Equation 15) $$\text{fold-change}(c=0)={\left(1+\frac{1}{1+e^{-\beta \Delta \varepsilon_{AI}}}\frac{R}{N_{NS}}e^{-\beta \Delta \varepsilon_{RA}}\right)}^{-1},$$

since it is missing the factor $\frac{1}{1+e^{-\beta \Delta \varepsilon_{AI}}}$ which specifies what fraction of repressors are in the active state in the absence of inducer,

(Equation 16) $$\frac{1}{1+e^{-\beta \Delta \varepsilon_{AI}}}=p_{A}(0).$$

In other words, Garcia and Phillips assumed that in the absence of inducer, all repressors were active. In terms of our notation, the convoluted energy values Δ*ε̃_RA_* extracted by Garcia and Phillips (namely, for O1 and for Oid) represent

(Equation 17) $$\beta \Delta {\tilde{\varepsilon }}_{RA}=\beta \Delta \varepsilon_{RA}-\log\left(\frac{1}{1+e^{-\beta \Delta \varepsilon_{AI}}}\right).$$

Note that if *e*^−*β*Δ*ε*_*AI*_^ ≪ 1, then nearly all of the repressors are active in the absence of inducer so that Δ*ε̃_RA_* ≈ Δ*ε_RA_*. In simple repression systems where we definitively know the value of Δε*_RA_* and *R*, we can use Equation 15 to determine the value of Δε*_AI_* by comparing with experimentally determined fold-change values. However, the binding energy values that we use from Garcia and Phillips (2011) are effective parameters Δ*ε̃_RA_*. In this case, we are faced with an undetermined system in which we have more variables than equations, and we are thus unable to determine the value of Δε*_AI_*. In order to obtain this parameter, we must turn to a more complex regulatory scenario which provides additional constraints that allow us to fit for Δε*_AI_*.

A variation on simple repression in which multiple copies of the promoter are available for repressor binding (for instance, when the simple repression construct is on plasmid) can be used to circumvent the problems that arise when using Δ*ε̃_RA_*. This is because the behavior of the system is distinctly different when the number of active repressors *p_A_*(0)*R* is less than or greater than the number of available promoters *N*. Repression data for plasmids with known copy number *N* allows us to perform a fit for the value of Δε*_AI_*.

To obtain an expression for a system with multiple promoters *N*, we follow Weinert et al. (2014), writing the fold-change in terms of the the grand canonical ensemble as

(Equation 18) $$\text{fold-change}=\frac{1}{1+\lambda_{r}e^{-\beta \Delta \varepsilon_{RA}}},$$

where *λ_r_*=*e^βμ^* is the fugacity and *μ* is the chemical potential of the repressor. The fugacity will enable us to easily enumerate the possible states available to the repressor.

To determine the value of *λ_r_*, we first consider that the total number of repressors in the system, *R*_tot_, is fixed and given by

(Equation 19) $$R_{\text{tot}}=R_{S}+R_{NS},$$

where *R_S_* represents the number of repressors specifically bound to the promoter and *R_NS_* represents the number of repressors nonspecifically bound throughout the genome. The value of *R_S_* is given by

(Equation 20) $$R_{S}=N\frac{\lambda_{r}e^{-\beta \Delta \varepsilon_{RA}}}{1+\lambda_{r}e^{-\beta \Delta \varepsilon_{RA}}},$$

where *N* is the number of available promoters in the cell. Note that in counting *N*, we do not distinguish between promoters that are on plasmid or chromosomally integrated provided that they both have the same repressor-operator binding energy (Weinert et al., 2014). The value of *R_NS_* is similarly give by

(Equation 21) $$R_{NS}=N_{NS}\frac{\lambda_{r}}{1+\lambda_{r}},$$

where *N_NS_* is the number of non-specific sites in the cell (recall that we use *N_NS_* = 4.6 × 10^6^ for *E. coli*).

Substituting in Equations 20 and 21 into the modified Equation 19 yields the form

(Equation 22) $$p_{A}(0)R_{\text{tot}}=\frac{1}{1+e^{-\beta \Delta \varepsilon_{AI}}}\left(N\frac{\lambda_{r}e^{-\beta \Delta \varepsilon_{RA}}}{1+\lambda_{r}e^{-\beta \Delta \varepsilon_{RA}}}+N_{NS}\frac{\lambda_{r}}{1+\lambda_{r}}\right),$$

where we recall from Equation 17 that $\beta \Delta \varepsilon_{RA}=\beta \Delta {\tilde{\varepsilon }}_{RA}+\log\left(\frac{1}{1+e^{-\beta \Delta \varepsilon_{AI}}}\right)$. Numerically solving for *λ_r_* and plugging the value back into Equation 18 yields a fold-change function in which the only unknown parameter is Δε*_AI_*.

With these calculations in hand, we can now determine the value of the Δε*_AI_* parameter. Figure S5A shows how different values of Δε*_AI_* lead to significantly different fold-change response curves. Thus, analyzing the specific fold-change response of any strain with a known plasmid copy number *N* will fix Δε*_AI_*. Notably, the inflection point of Equation 22 occurs near *p_A_*(0)*R*_tot_ = *N* (as shown by the triangles in Figure S5A), so that merely knowing where the fold-change response transitions from concave down to concave up is sufficient to obtain a rough value for Δε*_AI_*. We note, however, that for Δ*ε_AI_* ≳ 5 *k_B_T*, increasing Δε*_AI_* further does not affect the fold-change because essentially every repressor will be in the active state in this regime. Thus, if the Δε*_AI_* is in this regime, we can only bound it from below.

We now analyze experimental induction data for different strains with known plasmid copy numbers to determine Δε*_AI_*. Figure S5B shows experimental measurements of fold-change for two O1 promoters with *N* = 64 and *N* = 52 copy numbers and one Oid promoter with *N* = 10 from Brewster et al. (2014). By fitting these data to Equation 18, we extracted the parameter value Δ*ε_AI_* = 4.5 *k_B_T*. Substituting this value into Equation 16 shows that 99% of the repressors are in the active state in the absence of inducer and Δ*ε̃_RA_* ≈ Δ*ε_RA_*, so that all of the previous energies and calculations made by Garcia and Phillips (2011; Brewster et al., 2014) were accurate.

#### Alternate Characterizations of Induction

In this section we discuss a different way to describe the induction data, namely, through using the conventional Hill approach. We first demonstrate how using a Hill function to characterize a single induction curve enables us to extract features (such as the midpoint and sharpness) of that single response, but precludes any predictions of the other seventeen strains. We then discuss how a thermodynamic model of simple repression coupled with a Hill approach to the induction response can both characterize an induction profile and predict the response of all eighteen strains, although we argue that such a description provides no insight into the allosteric nature of the protein and how mutations to the repressor would affect induction. We conclude the section by discussing the differences between such a model and the statistical mechanical model used in the main text.

##### Fitting Induction Curves Using a Hill Function Approach

The Hill equation is a phenomenological function commonly used to describe data with a sigmoidal profile (Murphy et al., 2007; Murphy et al., 2010; Rogers et al., 2015). Its simplicity and ability to estimate the cooperativity of a system (through the Hill coefficient) has led to its widespread use in many domains of biology (Frank, 2013). Nevertheless, the Hill function is often criticized as a physically unrealistic model and the extracted Hill coefficient is often difficult to contextualize in the physics of a system (Weiss, 1997). In the present work, we note that a Hill function, even if it is only used because of its simplicity, presents no mechanism to understand how a regulatory system’s behavior will change if physical parameters such as repressor copy number or operator binding energy are varied. In addition, the Hill equation provides no foundation to explore how mutating the repressor (e.g., at its inducer-binding interface) would modify its induction profile, although statistical mechanical models have proved capable of characterizing such scenarios (Keymer et al., 2006; Swem et al., 2008; Einav et al., 2016).

Consider the general Hill equation for a single induction profile given by

(Equation 23) $$\text{fold-change}=(\text{leakiness})+(\text{dynamic}\;\text{range})\frac{{\left(\frac{c}{K}\right)}^{n}}{1+{\left(\frac{c}{K}\right)}^{n}},$$

where, as in the main text, the leakiness represents the minimum fold-change, the dynamic range represents the difference between the maximum and minimum fold-change, *K* is the repressor-inducer dissociation constant, and *n* denotes the Hill coefficient that characterizes the sharpness of the curve (*n* > 1 signifies positive cooperativity, *n* = 1 denotes no cooperativity, and *n* < 1 represents negative cooperativity). Figure S6 shows how the individual induction profiles can be fit (using the same Bayesian methods as described in Section “Global Fit of All Parameters”) to this Hill response, yielding a similar response to that shown in Figure 4D. However, characterizing the induction response in this manner is unsatisfactory because each curve must be fit independently thus removing our predictive power for other repressor copy numbers and binding sites.

The fitted parameters obtained from this approach are shown in Figure S7. These are rather unsatisfactory because they do not clearly reflect the properties of the physical system under consideration. For example, the dissociation constant *K* between LacI and inducer should not be affected by either the copy number of the repressor or the DNA binding energy, and yet we see upward trends as *R* is increased or the binding energy is decreased. Here, the *K* parameter ultimately describes the midpoint of the induction curve and therefore cannot strictly be considered a dissociation constant. Similarly, the Hill coefficient *n* does not directly represent the cooperativity between the repressor and the inducer as the molecular details of the copy number and DNA binding strength are subsumed in this parameter as well. While the leakiness and dynamic range describe important phenotypic properties of the induction response, this Hill approach leaves us with no means to predict them for other strains. In summary, the Hill equation Equation 23 cannot predict how an induction profile varies with repressor copy number, operator binding energy, or how mutations will alter the induction profile. To that end, we turn to a more sophisticated approach where we use the Hill function to describe the available fraction of repressor as a function of inducer concentration.

##### Fitting Induction Curves Using a Combination Thermodynamic Model and Hill Function Approach

Motivated by the inability in the previous section to characterize all eighteen strains using the Hill function with a single set of parameters, here we combine the Hill approach with a thermodynamic model of simple repression to garner predictive power. More specifically, we will use the thermodynamic model in Figure 2A but substitute the statistical model in Figure 2B with the phenomenological Hill function Equation 23.

Following Equations 1, 2, and 3, fold-change is given by

(Equation 24) $$\text{fold-change}={\left(1+p_{A}(c)\frac{R}{N_{NS}}e^{-\beta \Delta \varepsilon_{RA}}\right)}^{-1},$$

where the Hill function

(Equation 25) $$p_{A}(c)=p_{A}^{\max}-p_{A}^{\text{range}}\frac{{\left(\frac{c}{K_{D}}\right)}^{n}}{1+{\left(\frac{c}{K_{D}}\right)}^{n}},$$

represents the fraction of repressors in the allosterically active state, with $p_{A}^{\max}$ denoting the fraction of active repressors in the absence of inducer and $p_{A}^{\max}-p_{A}^{\text{range}}$ the minimum fraction of active repressors in the presence of saturating inducer. The Hill function characterizes the inducer-repressor binding while the thermodynamic model with the known constants *R*, *N_NS_*, and Δε*_RA_* describes how the induction profile changes with repressor copy number and repressor-operator binding energy.

As in the main text, we can fit the four Hill parameters – the vertical shift and stretch parameters $p_{A}^{\max}$ and $p_{A}^{\text{range}}$, the Hill coefficient *n*, and the inducer-repressor dissociation constant *K_D_* – for a single induction curve and then use the fully characterized Equation 24 to describe the response of each of the eighteen strains. Figure S8 shows this process carried out by fitting the O2 *R* = 260 strain (white circles in [B]) and predicting the behavior of the remaining seventeen strains.

Although the curves in Figure S8 are nearly identical to those in Figure 4 (which were made using the MWC model Equation 5), we stress that the Hill function approach is more complex than the MWC model (containing four parameters instead of three) and it obscures the relationships to the physical parameters of the system. For example, it is not clear whether the fit parameter $K_{D}=4_{-1}^{+2}\times 10^{-6}\;M$ relays the dissociation constant between the inducer and active-state repressor, between the inducer and the inactive-state repressor, or some mix of the two quantities.

In addition, the MWC model Equation 5 naturally suggests further quantitative tests for the fold-change relationship. For example, mutating the repressor’s inducer binding site would likely alter the repressor-inducer dissociation constants *K_A_* and *K_I_*, and it would be interesting to find out if such mutations also modify the allosteric energy difference Δε*_AI_* between the repressor’s active and inactive conformations. For our purposes, the Hill function Equation 25 falls short of the connection to the physics of the system and provides no intuition about how transcription depends upon such mutations. For these reasons, we present the thermodynamic model coupled with the statistical mechanical MWC model approach in the paper.

##### Global Fit of all Parameters

In the main text, we used the repressor copy numbers *R* and repressor-DNA binding energies Δε*_RA_* as reported by Garcia and Phillips (2011). However, any error in these previous measurements of *R* and Δε*_RA_* will necessarily propagate into our own fold-change predictions. In this section we take an alternative approach to fitting the physical parameters of the system to that used in the main text. First, rather than fitting only a single strain, we fit the entire data set in Figure 5 along with microscopy data for the synthetic operator Oid (see Appendix D accessible through https://doi.org/10.22002/D1.743). In addition, we also simultaneously fit the parameters *R* and Δε*_RA_* using the prior information given by the previous measurements. By using the entire data set and fitting all of the parameters, we obtain the best possible characterization of the statistical mechanical parameters of the system given our current state of knowledge. As a point of reference, we state all of the parameters of the MWC model derived in the text in Table S3.

To fit all of the parameters simultaneously, we follow a similar approach to the one detailed in the Quantification and Statistical Analysis section. Briefly, we perform a Bayesian parameter estimation of the dissociation constants *K_A_* and *K_I_*, the six different repressor copy numbers *R* corresponding to the six *lacI* ribosomal binding sites used in our work, and the four different binding energies Δε*_RA_* characterizing the four distinct operators used to make the experimental strains. As in the main text, we fit the logarithms ${\tilde{k}}_{A}=-\log\frac{K_{A}}{1\;M}$ and ${\tilde{k}}_{I}=-\log\frac{K_{I}}{1\;M}$ of the dissociation constants which grants better numerical stability.

As in Equations 24 and 25, we assume that deviations of the experimental fold-change from the theoretical predictions are normally distributed with mean zero and standard deviation *σ*. We begin by writing Bayes’ theorem,

(Equation 26) $$P\left({\tilde{k}}_{A},{\tilde{k}}_{I},R,\Delta \varepsilon_{\mathrm{RA}},\sigma |D\right)=\frac{P\left(D|{\tilde{k}}_{A},{\tilde{k}}_{I},R,\Delta \varepsilon_{\mathrm{RA}},\sigma \right)P\left({\tilde{k}}_{A},{\tilde{k}}_{I},R,\Delta \varepsilon_{\mathrm{RA}},\sigma \right)}{P(D)},$$

where ***R*** is an array containing the six different repressor copy numbers to be fit, **Δ**ε***_RA_*** is an array containing the four binding energies to be fit, and *D* is the experimental fold-change data. The term *P* (*k̃_A_*, *k̃_I_*, *R*, **Δ**ε***_RA_***, *σ*|*D*) gives the probability distributions of all of the parameters given the data. The term *P* (D|*k̃_A_*, *k̃_I_*, ***R***, **Δ**ε***_RA_***, *σ*) represents the likelihood of having observed our experimental data given some value for each parameter. *P* (*k̃_A_*, *k̃_I_*, ***R***, **Δ**ε***_RA_***, *σ*) contains all the prior information on the values of these parameters. Lastly, *P*(*D*) serves as a normalization constant and hence can be ignored.

Given *n* independent measurements of the fold-change, the first term in can be written as

(Equation 27) $$P\left(D|{\tilde{k}}_{A},{\tilde{k}}_{I},R,\Delta \varepsilon_{RA},\sigma \right)=\frac{1}{{\left(2\pi \sigma^{2}\right)}^{\frac{n}{2}}}\prod_{i=1}^{n}\exp\;\left[-\frac{{\left(fc_{\exp}^{\left(i\right)}-fc\;\left({\tilde{k}}_{A},{\tilde{k}}_{I},R^{\left(i\right)},\Delta \varepsilon_{RA}^{\left(i\right)},c^{\left(i\right)}\right)\right)}^{2}}{2\sigma^{2}}\right],$$

where ${\text{fc}}_{\exp}^{\left(i\right)}$ is the *i*^th^ experimental fold-change and fc(•••) is the theoretical prediction. Note that the standard deviation *σ* of this distribution is not known and hence needs to be included as a parameter to be fit.

The second term in represents the prior information of the parameter values. We assume that all parameters are independent of each other, so that

(Equation 28) $$P\left({\tilde{k}}_{A},{\tilde{k}}_{I},R,\Delta \varepsilon_{RA},\sigma \right)=P\left({\tilde{k}}_{A}\right)•P\left({\tilde{k}}_{I}\right)•\prod_{i}P(R^{\left(i\right)})•\prod_{j}P\left(\Delta \varepsilon_{RA}^{\left(j\right)}\right)•P(\sigma ),$$

where the superscript (*i*) indicates the repressor copy number of index *i* and the superscript (*j*) denotes the binding energy of index *j*. As above, we note that a prior must also be included for the unknown parameter *σ*.

Because we knew nothing about the values of *k̃_A_*, *k̃_I_*, and *σ* before performing the experiment, we assign maximally uninformative priors to each of these parameters. More specifically, we assign uniform priors to *k̃_A_* and *k̃_I_* and a Jeffreys prior to *σ*, indicating that *K_A_*, *K_I_*, and *σ* are scale parameters (Sivia and Skilling, 2006). We do, however, have prior information for the repressor copy numbers and the repressor-DNA binding energies from Garcia and Phillips (2011). This prior knowledge is included within our model using an informative prior for these two parameters, which we assume to be Gaussian. Hence each of the *R*^(^*^i^*^)^ repressor copy numbers to be fit satisfies

(Equation 29) $$P(R^{\left(i\right)})=\frac{1}{\sqrt{2\pi \sigma_{R_{i}}^{2}}}\exp\;\left(-\frac{{\left(R^{\left(i\right)}-{\bar{R}}^{\left(i\right)}\right)}^{2}}{2\sigma_{R_{i}}^{2}}\right),$$

where *R̄*^(^*^i^*^)^ is the mean repressor copy number and *σ*_*R*_*i*__ is the variability associated with this parameter as reported in Garcia and Phillips (2011). Note that we use the given value of *σ*_*R*_*i*__ from previous measurements rather than leaving this as a free parameter.

Similarly, the binding energies $\Delta \varepsilon_{RA}^{\left(j\right)}$ are also assumed to have a Gaussian informative prior of the same form. We write it as

(Equation 30) $$P\left(\Delta \varepsilon_{RA}^{\left(j\right)}\right)=\frac{1}{\sqrt{2\pi \sigma_{\varepsilon_{j}}^{2}}}\exp\;\left(-\frac{{\left(\Delta \varepsilon_{RA}^{\left(j\right)}-\Delta {\bar{\varepsilon }}_{RA}^{\left(j\right)}\right)}^{2}}{2\sigma_{\varepsilon_{j}}^{2}}\right),$$

where $\Delta {\bar{\varepsilon }}_{RA}^{\left(j\right)}$ is the binding energy and *σ*_*ε*_*j*__ is the variability associated with that parameter around the mean value as reported in Garcia and Phillips (2011).

The *σ*_*R*_*i*__ and *σ*_*ε*_*j*__ parameters will constrain the range of values for *R*^(^*^i^*^)^ and $\Delta \varepsilon_{RA}^{\left(j\right)}$ found from the fitting. For example, if for some *i* the standard deviation *σ*_*R*_*i*__ is very small, it implies a strong confidence in the previously reported value. Mathematically, the exponential in Equation 29 will ensure that the best-fit *R*^(^*^i^*^)^ lies within a few standard deviations of *R̄*^(^*^i^*^)^. Since we are interested in exploring which values could give the best fit, the errors are taken to be wide enough to allow the parameter estimation to freely explore parameter space in the vicinity of the best estimates. Putting all these terms together, we use Markov chain Monte Carlo to sample the posterior distribution *P* (*k̃_A_*, *k̃_I_*, ***R***, **Δ**ε***_RA_***, *σ*|*D*), enabling us to determine both the most likely value for each physical parameter as well as its associated credible region (see the GitHub repository (http://doi.org/10.5281/zenodo.1163620) for the implementation).

Figure S9 shows the result of this global fit. When compared with Figure 5 we can see that fitting for the binding energies and the repressor copy numbers improves the agreement between the theory and the data. Table S4 summarizes the values of the parameters as obtained with this MCMC parameter inference. We note that even though we allowed the repressor copy numbers and repressor-DNA binding energies to vary, the resulting fit values were very close to the previously reported values. The fit values of the repressor copy numbers were all within one standard deviation of the previous reported values provided in Garcia and Phillips (2011). And although some of the repressor-DNA binding energies differed by a few standard deviations from the reported values, the differences were always less than 1 *k_B_T*, which represents a small change in the biological scales we are considering. The biggest discrepancy between our fit values and the previous measurements arose for the synthetic Oid operator, which we discuss in more detail in Appendix D accessible through https://doi.org/10.22002/D1.743.

Figure S10 shows the same key properties as in Figure 6, but uses the parameters obtained from this global fitting approach. We note that even by increasing the number of degrees of freedom in our fit, the result does not change substantially, due to in general, only minor improvements between the theoretical curves and data. For the O3 operator data, again, agreement between the predicted [EC_50_] and the effective Hill coefficient remain poor due the theory being unable to capture the steepness of the response curves.

#### Comparison of Parameter Estimation and Fold-Change Predictions across Strains

The inferred parameter values for *K_A_* and *K_I_* in the main text were determined by fitting to induction fold-change measurements from a single strain (*R* = 260, Δ*ε_RA_* = −13.9 *k_B_T*, *n*=2, and Δ*ε_AI_* = 4.5 *k_B_T*). After determining these parameters, we were able to predict the fold-change of the remaining strains without any additional fitting. However, the theory should be independent of the specific strain used to estimate *K_A_* and *K_I_*; using any alternative strain to fit *K_A_* and *K_I_* should yield similar predictions. For the sake of completeness, here we discuss the values for *K_A_* and *K_I_* that are obtained by fitting to each of the induction data sets individually. These fit parameters are shown in Figure 5D of the main text, where we find close agreement between strains, but with some deviation and poorer inferences observed with the O3 operator strains. Overall, we find that regardless of which strain is chosen to determine the unknown parameters, the predictions laid out by the theory closely match the experimental measurements. Here we present a comparison of the strain specific predictions and measured fold-change data for each of the three operators considered.

We follow the approach taken in the main text and use Equation 5 to infer values for *K_A_* and *K_I_* by fitting to each combination of binding energy *Δ*ε*_RA_* and repressor copy number *R*. We then use these fitted parameters to predict the induction curves of all other strains. In Figure S11 we plot these fold-change predictions along with experimental data for each of our strains that contains an O1 operator. To make sense of this plot consider the first row as an example. In the first row, *K_A_* and *K_I_* were estimated using data from the strain containing *R*=22 and an O1 operator (top leftmost plot, shaded in gray). The remaining plots in this row show the predicted fold-change using these values for *K_A_* and *K_I_*. In each row, we then infer *K_A_* and *K_I_* using data from a strain containing a different repressor copy number (*R* = 60 in the second row, *R* = 124 in the third row, and so on). In Figures S12 and S13, we similarly apply this inference to our strains with O2 and O3 operators, respectively. We note that the overwhelming majority of predictions closely match the experimental data. The notable exception is that using the *R* = 22 strain provides poor predictions for the strains with large copy numbers (especially *R* = 1220 and *R* = 1740), though it should be noted that predictions made from the *R* = 22 strain have considerably broader credible regions. This loss in predictive power is due to the poorer estimates of *K_A_* and *K_I_* for the *R* = 22 strain as shown in Figure 5D.

#### Properties of Induction Titration Curves

In this section, we expand on the phenotypic properties of the induction response that were explored in the main text (see Figure 1). We begin by expanding on our discussion of dynamic range and then show the analytic form of the [EC_50_] for simple repression.

As stated in the main text, the dynamic range is defined as the difference between the maximum and minimum system response, or equivalently, as the difference between the saturation and leakiness of the system. Using Equations 6, 7, and 8, the dynamic range is given by

(Equation 31) $$\text{dynamic}\;\text{range}={\left(1+\frac{1}{1+e^{-\beta \Delta \varepsilon_{AI}}\;{\left(\frac{K_{A}}{K_{I}}\right)}^{n}}\frac{R}{N_{NS}}e^{-\beta \Delta \varepsilon_{RA}}\right)}^{-1}-{\left(1+\frac{1}{1+e^{-\beta \Delta \varepsilon_{AI}}}\frac{R}{N_{NS}}e^{-\beta \Delta \varepsilon_{RA}}\right)}^{-1}.$$

The dynamic range, along with saturation and leakiness were plotted with our experimental data in Figures 6A–6C as a function of repressor copy number. Figure S14 shows how these properties are expected to vary as a function of the repressor-operator binding energy. Note that the resulting curves for all three properties have the same shape as in Figures 6A–6C, since the dependence of the fold-change upon the repressor copy number and repressor-operator binding energy are both contained in a single multiplicative term, *Re*^−*β*Δ*ε*_*RA*_^. Hence, increasing *R* on a logarithmic scale (as in Figures 6A–6C) is equivalent to decreasing Δε*_RA_* on a linear scale (as in Figure S14).

An interesting aspect of the dynamic range is that it exhibits a peak as a function of either the repressor copy number (or equivalently of the repressor-operator binding energy). Differentiating the dynamic range Equation 31 and setting it equal to zero, we find that this peak occurs at

(Equation 32) $$\frac{R^{*}}{N_{NS}}=e^{-\beta (\Delta \varepsilon_{AI}-\Delta \varepsilon_{RA})}\sqrt{e^{\Delta \varepsilon_{AI}}+1}\sqrt{e^{\Delta \varepsilon_{AI}}+{\left(\frac{K_{A}}{K_{I}}\right)}^{n}}.$$

The magnitude of the peak is given by

(Equation 33) $$\max\text{dynamic}\;\text{range}=\frac{{\left(\sqrt{e^{\Delta \varepsilon_{AI}}+1}-\sqrt{e^{\Delta \varepsilon_{AI}}+{\left(\frac{K_{A}}{K_{I}}\right)}^{n}}\right)}^{2}}{{\left(\frac{K_{A}}{K_{I}}\right)}^{n}-1},$$

which is independent of the repressor-operator binding energy Δε*_RA_* or *R*, and will only cause a shift in the location of the peak but not its magnitude.

We now consider the two remaining properties, the [EC_50_] and effective Hill coefficient, which determine the horizontal properties of a system - that is, they determine the range of inducer concentration in which the system’s response goes from its minimum to maximum values. The [EC_50_] denotes the inducer concentration required to generate fold-change halfway between its minimum and maximum value and was defined implicitly in Equation 9. For the simple repression system, the [EC_50_] is given by

(Equation 34) $$\frac{\left[{\text{EC}}_{50}\right]}{K_{A}}=\frac{\frac{K_{A}}{K_{I}}-1}{\frac{K_{A}}{K_{I}}-{\left(\frac{\left(1+\frac{R}{N_{NS}}e^{-\beta \Delta \varepsilon_{RA}}\right)+{\left(\frac{K_{A}}{K_{I}}\right)}^{n}\left(2e^{-\beta \Delta \varepsilon_{AI}}+\left(1+\frac{R}{N_{NS}}e^{-\beta \Delta \varepsilon_{RA}}\right)\right)}{2\left(1+\frac{R}{N_{NS}}e^{-\beta \Delta \varepsilon_{RA}}\right)+e^{-\beta \Delta \varepsilon_{AI}}+{\left(\frac{K_{A}}{K_{I}}\right)}^{n}e^{-\beta \Delta \varepsilon_{AI}}}\right)}^{\frac{1}{n}}}-1.$$

Using this expression, we can then find the effective Hill coefficient *h*, which equals twice the log-log slope of the normalized fold-change evaluated at *c* = [EC_50_] (see Equation 10). In Figures 6D and 6E we show how these two properties vary with repressor copy number, and in Figure S15 we demonstrate how they depend on the repressor-operator binding energy. Both the [EC_50_] and *h* vary significantly with repressor copy number for sufficiently strong operator binding energies. Notably, for weak operator binding energies on the order of the O3 operator, it is predicted that the effective Hill coefficient should not vary with repressor copy number. In addition, the maximum possible Hill coefficient is roughly 1.75, which stresses the point that the effective Hill coefficient should not be interpreted as the number of inducer binding sites, which is exactly 2.

### QUANTIFICATION AND STATISTICAL ANALYSIS

In this work, we determine the most likely parameter values for the inducer dissociation constants *K_A_* and *K_I_* of the active and inactive state, respectively, using Bayesian methods. We compute the probability distribution of the value of each parameter given the data *D*, which by Bayes’ theorem is given by

(Equation 35) $$P(K_{A},K_{I}|D)=\frac{P(D|K_{A},K_{I})P(K_{A},K_{I})}{P(D)},$$

where *D* is all the data composed of independent variables (repressor copy number *R*, repressor-DNA binding energy Δε*_RA_*, and inducer concentration *c*) and one dependent variable (experimental fold-change). *P*(*D*|*K_A_*, *K_I_*) is the likelihood of having observed the data given the parameter values for the dissociation constants, *P* (*K_A_*, *K_I_*) contains all the prior information on these parameters, and *P* (*D*) serves as a normalization constant, which we can ignore in our parameter estimation. Equation 5 assumes a deterministic relationship between the parameters and the data, so in order to construct a probabilistic relationship as required by Equation 35, we assume that the experimental fold-change for the *i*^th^ datum given the parameters is of the form

(Equation 36) $$\text{fold}-{\text{change}}_{\exp}^{\left(i\right)}={\left(1+\frac{{\left(1+\frac{c^{\left(i\right)}}{K_{A}}\right)}^{2}}{{\left(1+\frac{c^{\left(i\right)}}{K_{A}}\right)}^{2}+e^{-\beta \Delta \varepsilon_{AI}}\;{\left(1+\frac{c^{\left(i\right)}}{K_{I}}\right)}^{2}}\frac{R^{\left(i\right)}}{N_{NS}}e^{-\beta \Delta \varepsilon_{RA}^{\left(i\right)}}\right)}^{-1}+\varepsilon^{\left(i\right)},$$

where *ε*^(^*^i^*^)^ represents the departure from the deterministic theoretical prediction for the *i*^th^ data point. If we assume that these *ε*^(^*^i^*^)^ errors are normally distributed with mean zero and standard deviation *σ*, the likelihood of the data given the parameters is of the form

(Equation 37) $$P(D|K_{A},K_{I},\sigma )=\frac{1}{{\left(2\pi \sigma^{2}\right)}^{\frac{n}{2}}}\prod_{i=1}^{n}\exp\;\left[-\frac{{\left(\text{fold}-{\text{change}}_{\exp}^{\left(i\right)}-\text{fold-change}\left(K_{A},K_{I},R^{\left(i\right)},\Delta \varepsilon_{RA}^{\left(i\right)},c^{\left(i\right)}\right)\right)}^{2}}{2\sigma^{2}}\right],$$

where $\text{fold}-{\text{change}}_{\exp}^{\left(i\right)}$ is the experimental fold-change and fold – change(···) is the theoretical prediction. The product $\Pi_{i=1}^{n}$ captures the assumption that the *n* data points are independent. Note that the likelihood and prior terms now include the extra unknown parameter *σ*. In applying Equation 37, a choice of *K_A_* and *K_I_* that provides better agreement between theoretical fold-change predictions and experimental measurements will result in a more probable likelihood.

Both mathematically and numerically, it is convenient to define ${\tilde{k}}_{A}=-\log\frac{K_{A}}{1\;M}$ and ${\tilde{k}}_{I}=-\log\frac{K_{I}}{1\;M}$ and fit for these parameters on a log scale. Dissociation constants are scale invariant, so that a change from 10 *μ*M to 1 *μ*M leads to an equivalent increase in affinity as a change from 1 *μ*M to 0.1 *μ*M. With these definitions we assume for the prior *P* (*k̃_A_*, *k̃_I_*, *σ*) that all three parameters are independent. In addition, we assume a uniform distribution for *k̃_A_* and *k̃_I_* and a Jeffreys prior (Sivia and Skilling, 2006) for the scale parameter *σ*. This yields the complete prior

(Equation 38) $$P\left({\tilde{k}}_{A},{\tilde{k}}_{I},\sigma \right)\equiv \frac{1}{\left({\tilde{k}}_{A}^{\max}-{\tilde{k}}_{A}^{\min}\right)}\frac{1}{\left({\tilde{k}}_{I}^{\max}-{\tilde{k}}_{I}^{\min}\right)}\frac{1}{\sigma }.$$

These priors are maximally uninformative meaning that they imply no prior knowledge of the parameter values. We defined the *k̃_A_* and *k̃_A_* ranges uniform on the range of −7 to 7, although we note that this particular choice does not affect the outcome provided the chosen range is sufficiently wide.

Putting all these terms together we can now sample from *P*(*k̃_A_*, *k̃_I_*, *σ*|*D*) using Markov chain Monte Carlo (see GitHub repository, http://doi.org/10.5281/zenodo.1163620) to compute the most likely parameter as well as the error bars (given by the 95% credible region) for *K_A_* and *K_I_*.

### DATA AND SOFTWARE AVAILABILITY

All of the data used in this work as well as all relevant code can be found at this dedicated website. Data were collected, stored, and preserved using the Git version control software in combination with off-site storage and hosting website GitHub. Code used to generate all figures and complete all processing step as and analyses are available on the GitHub repository. Many analysis files are stored as instructive Jupyter Notebooks. The scientific community is invited to fork our repositories and open constructive issues on the GitHub repository (http://doi.org/10.5281/zenodo.1163620).

## Supplementary Material

1

2

## Highlights

- The MWC model is used to understand allosteric transcription factor regulation
- Properties of predicted gene expression profiles are validated using LacI
- The data points collapse as a function of a key combinations of parameters
