# Supplementary material for: Tuning Transcriptional Regulation through Signaling: A Predictive Theory of Allosteric Induction
Source: Cell Syst. Author manuscript; Available in PMC 2018 Jun 7. (PMC5991102; doi:10.1016/j.cels.2018.02.004)
Supplement: 1 [file NIHMS964994-supplement-1.pdf]

Cell Systems, Volume *6*

Supplemental Information

Tuning Transcriptional Regulation through Signaling: A Predictive Theory of Allosteric Induction

Manuel Razo-Mejia, Stephanie L. Barnes, Nathan M. Belliveau, Griffin Chure, Tal Einav, Mitchell Lewis, and Rob Phillips

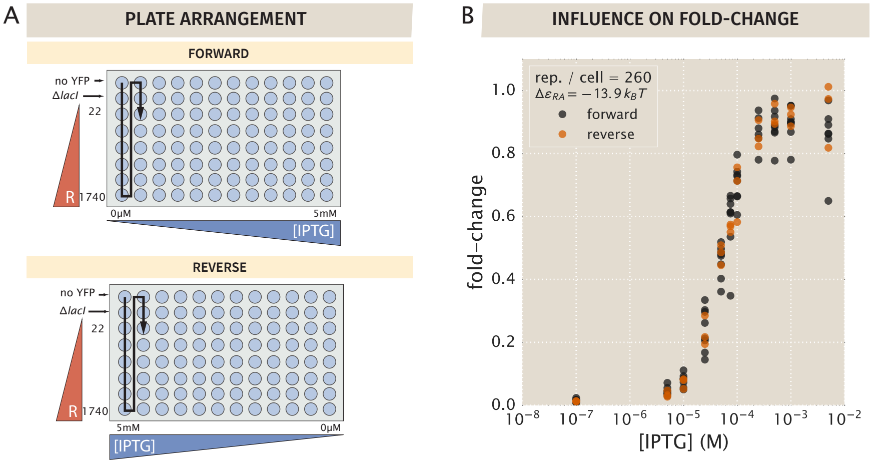

Figure S1. Plate arrangements for flow cytometry, related to STAR Methods. (A) Samples were measured primarily in the forward arrangement with a subset of samples measured in reverse. The black arrow indicates the order in which samples were processed by the flow cytometer. (B) The experimentally measured fold-change values for the two sets of plate arrangements show that samples measured in the forward arrangement appear to be indistinguishable from those measured in reverse order.

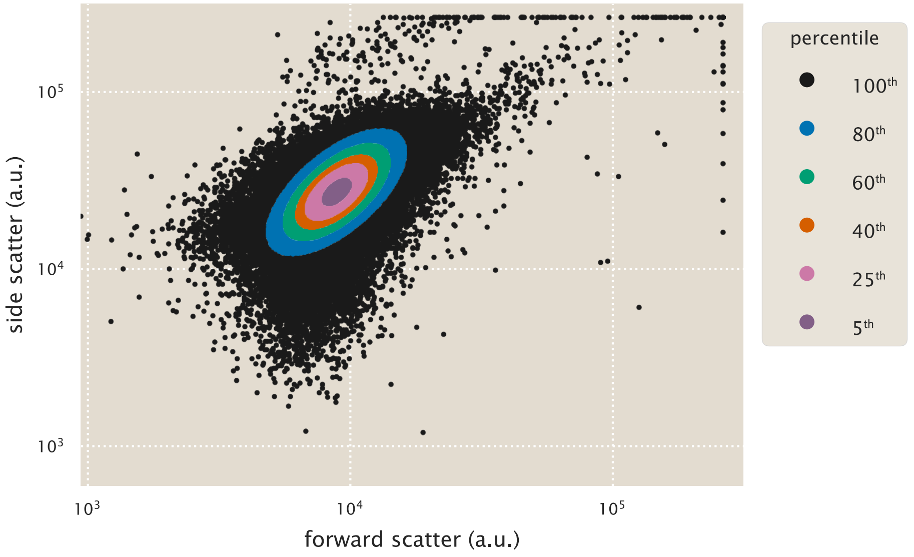

Figure S2. Representative unsupervised gating contours, related to STAR Methods. Points indicate individual flow cytometry measurements of forward scatter and side scatter. Colored points indicate arbitrary gating contours ranging from 100% ($\alpha = 1.0$) to 5% ($\alpha = 0.05$). All measurements for this work were made computing the mean fluorescence from the 40th percentile ($\alpha = 0.4$), shown as orange points.

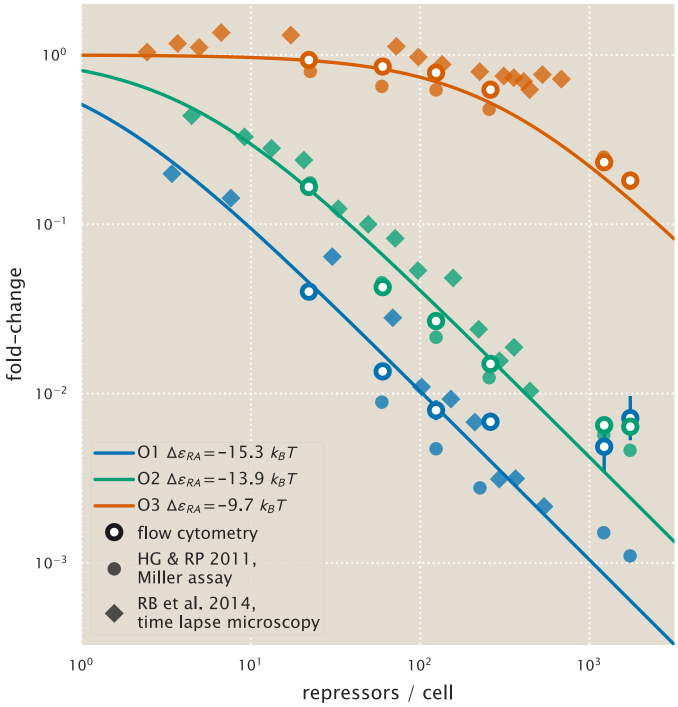

Figure S3. Comparison of experimental methods to determine the fold-change, related to STAR Methods. The fold-change in gene expression for equivalent simple-repression constructs has been determined using three independent methods: flow cytometry (this work), colorimetric Miller assays (Garcia and Phillips 2011a), and video microscopy (Brewster et al. 2014). All three methods give consistent results, although flow cytometry measurements lose accuracy for fold-change less than 10^{-2}. Note that the repressor-DNA binding energies $\Delta\varepsilon_{RA}$ used for the theoretical predictions were determined in Garcia and Phillips (2011a).

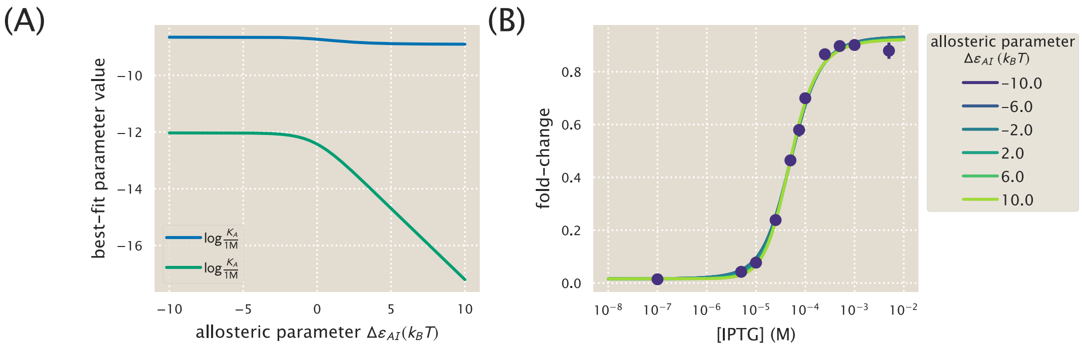

Figure S4. Multiple sets of parameters yield identical fold-change responses, related to STAR Methods. (A) The data for the O2 strain ($\Delta\varepsilon_{RA} = -13.9\ k_BT$) with $R = 260$ in Fig. 4(C) was fit using Eq. (5) with $n = 2$. $\Delta\varepsilon_{AI}$ is forced to take on the value shown on the x-axis, while the K_A and K_I parameters are fit freely. (B) The resulting best-fit functions for several value of $\Delta\varepsilon_{AI}$ all yield nearly identical fold-change responses.

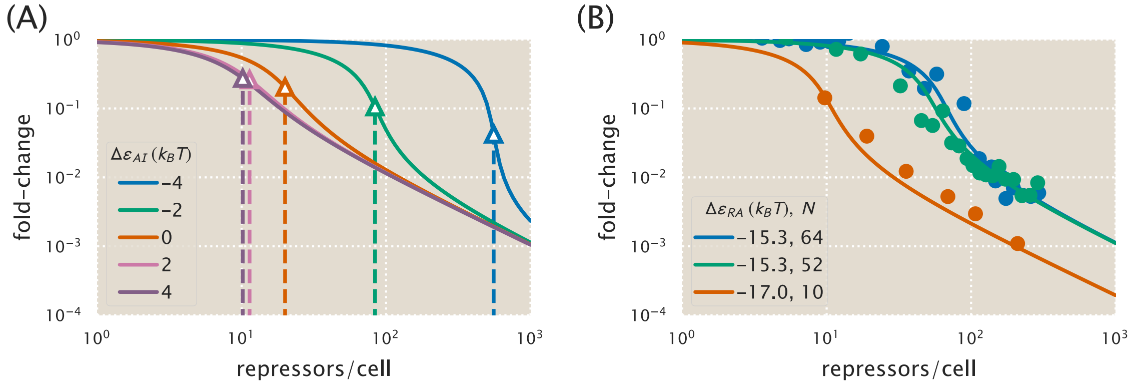

Figure S5. Fold-change of multiple identical genes, related to STAR Methods. (A) In the presence of $N = 10$ identical promoters, the fold-change Eq. (6) depends strongly on the allosteric energy difference $\Delta\varepsilon_{AI}$ between the Lac repressor's active and inactive states. The vertical dotted lines represent the number of repressors at which $R_A = N$ for each value of $\Delta\varepsilon_{AI}$. (B) Using fold-change measurements from (Brewster et al. 2014) for the operators and gene copy numbers shown, we can determine the most likely value $\Delta\varepsilon_{AI} = 4.5\ k_BT$ for LacI.

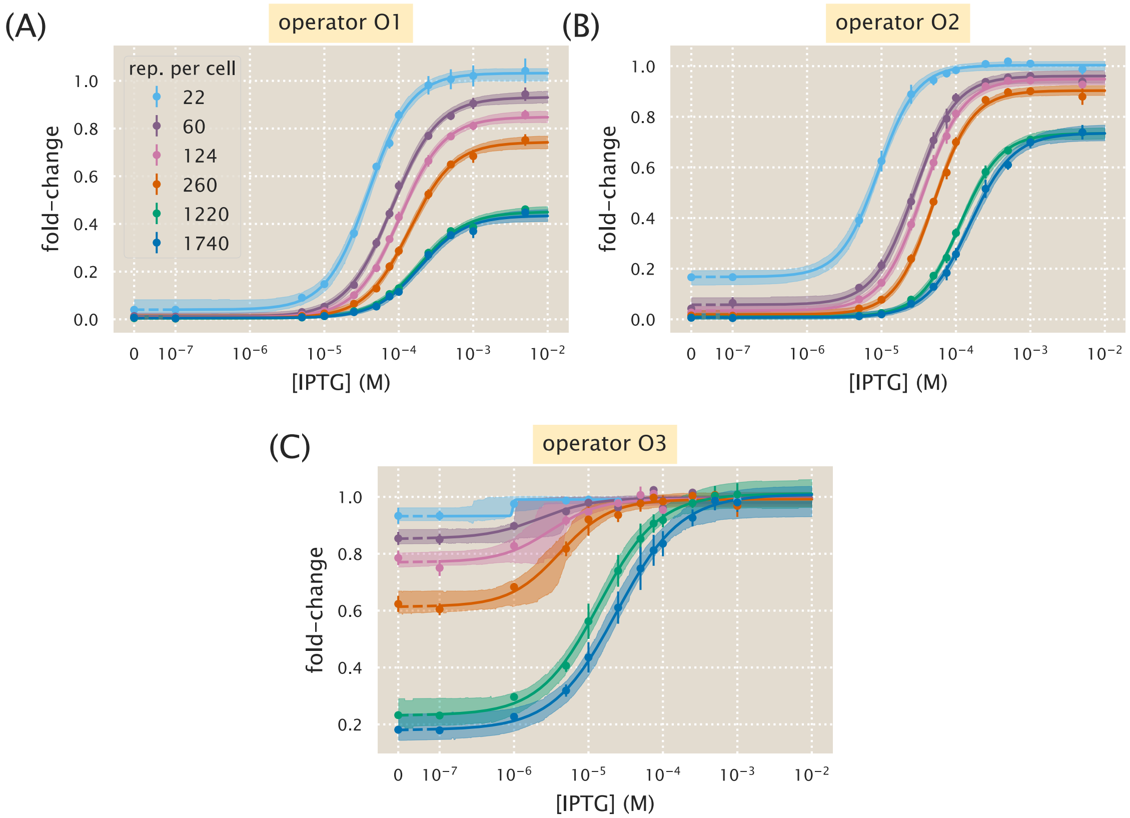

Figure S6. Hill function and MWC analysis of each induction profile, related to STAR Methods. Data for each individual strain was fit to the general Hill function in Eq. (11). (A) strains with O1 binding site, (B) strains with O2 binding site, and (C) strains with O3 binding site. Shaded regions indicate the bounds of the 95% credible region.

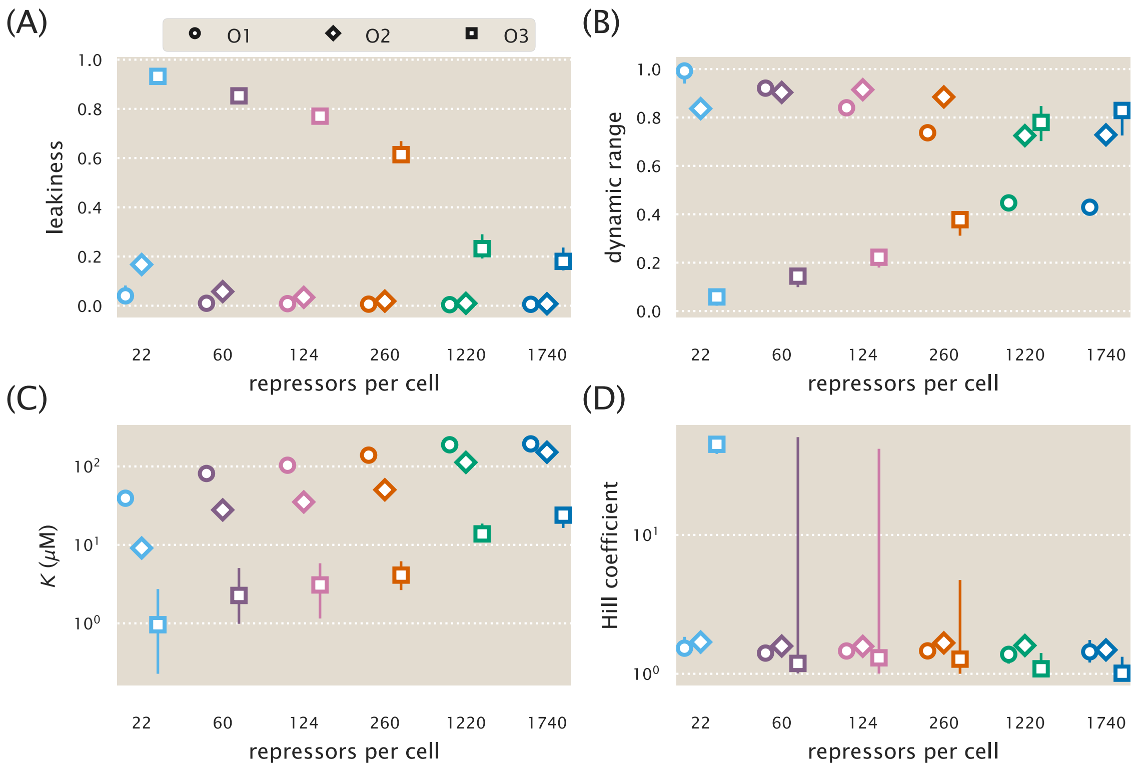

Figure S7. Parameter values for the Hill equation fit to each individual titration, related to STAR Methods. The resulting fit parameters from the Hill function fits of Fig. S6 are summarized. The large parameter intervals for many of the O3 strains are due to the flatter induction profile (as seen by its smaller dynamic range), and the ability for a large range of K and n values to describe the data.

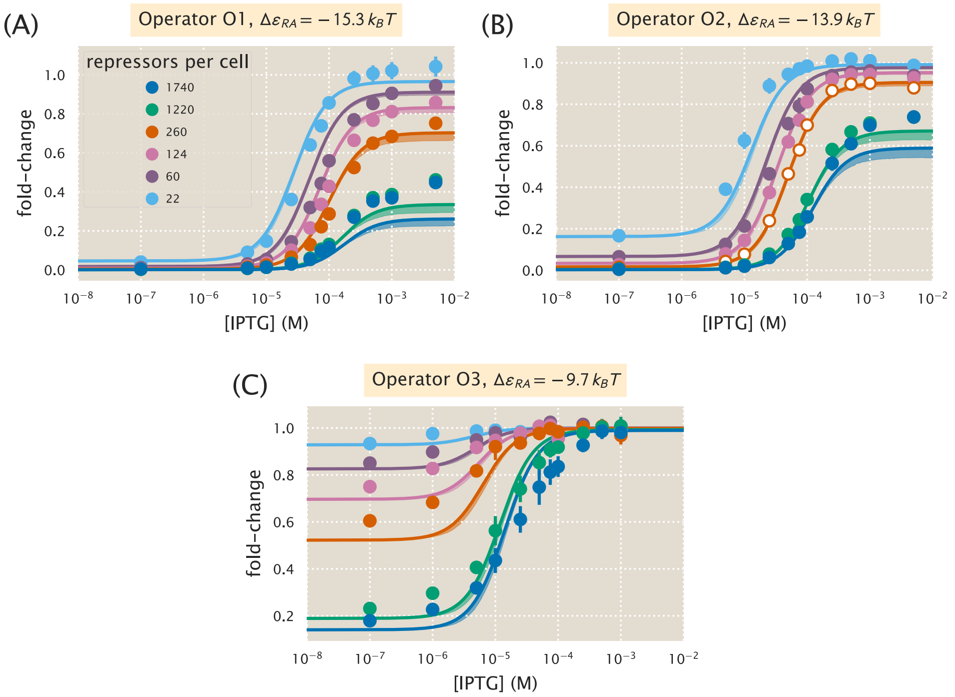

Figure S8. A thermodynamic model coupled with a Hill analysis can characterize induction, related to STAR Methods. Combining a thermodynamic model of simple repression with the Hill function to characterize the repressor-inducer binding successfully characterizes the induction profiles of all eighteen strains. As in the main text, data was only fit for the O2 $R = 260$ strain using Eqs. (12) and (13) and the parameters $p_A^{\max} = 0.90^{+0.03}_{-0.01}$, $p_A^{\text{range}} = -0.90^{+0.02}_{-0.03}$, $n = 1.6^{+0.2}_{-0.1}$, and $K_D = 4^{+2}_{-1} \times 10^{-6}$ M. Shaded regions indicate bounds of the 95% credible region.

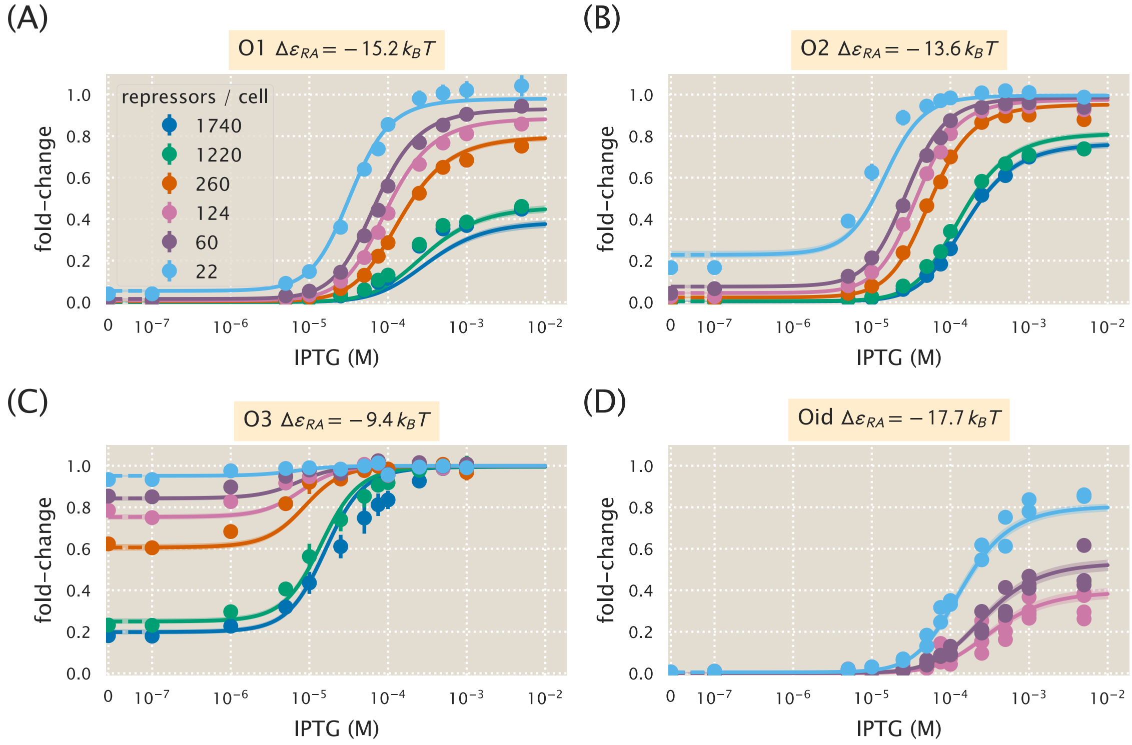

Figure S9. Global fit of dissociation constants, repressor copy numbers and binding energies, related to STAR Methods. Theoretical predictions resulting from simultaneously fitting the dissociation constants K_A and K_I, the six repressor copy numbers R, and the four repressor-DNA binding energies $\Delta\varepsilon_{RA}$ using the entire data set from Fig. 5 as well as the microscopy data for the Oid operator. Error bars of experimental data show the standard error of the mean (eight or more replicates) and shaded regions denote the 95% credible region. Where error bars are not visible, they are smaller than the point itself. For the Oid operator, all of the data points are shown since a smaller number of replicates were taken. The shaded regions are significantly smaller than in Fig. 5 because this fit was based on all data points, and hence the fit parameters are much more tightly constrained. The dashed lines at 0 IPTG indicates a linear scale, whereas solid lines represent a log scale.

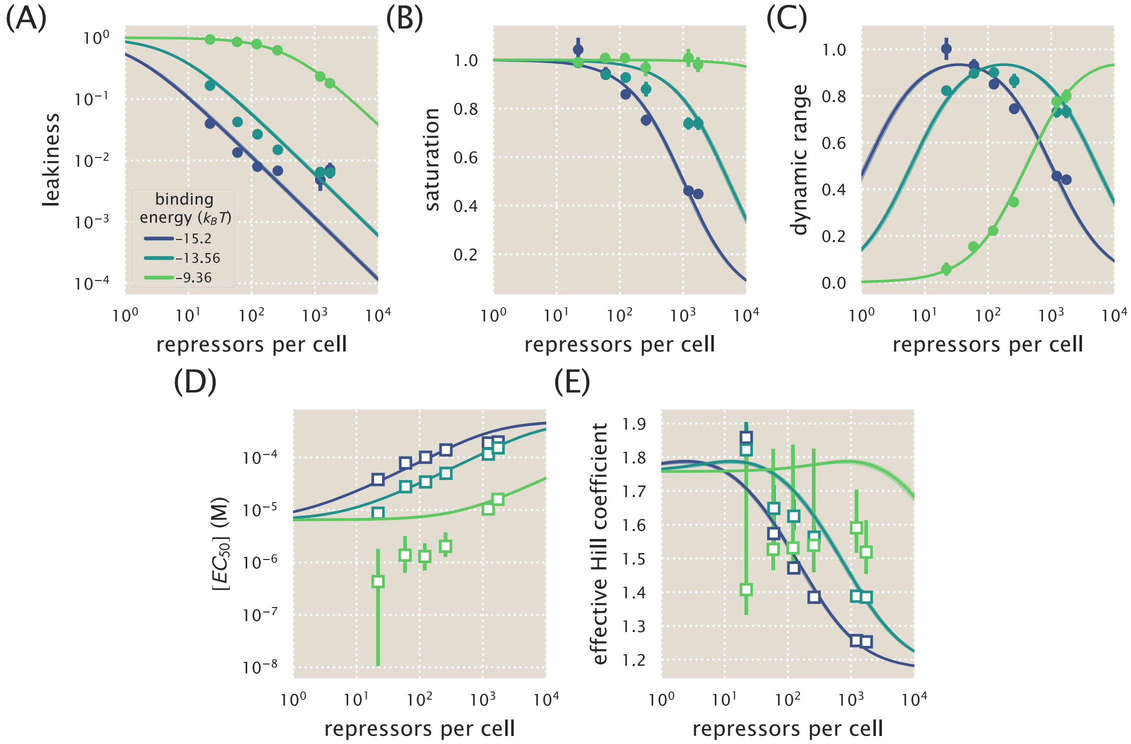

Figure S10. Key properties of induction profiles as predicted with a global fit using all available data, related to STAR Methods. Data for the (A) leakiness, (B) saturation, and (C) dynamic range are obtained from fold-change measurements in Fig. 5 in the absence and presence of IPTG. All prediction curves were generated using the parameters listed in S5. Both the (D) $[EC_{50}]$ and (E) effective Hill coefficient are inferred by individually fitting all parameters – K_A, K_I, R, $\Delta\varepsilon_{RA}$ – to each operator-repressor pairing in Fig. 5(A)-(C) separately to Eq. (5) in order to smoothly interpolate between the data points. Note that where error bars are not visible, this indicates that the error bars are smaller than the point itself.

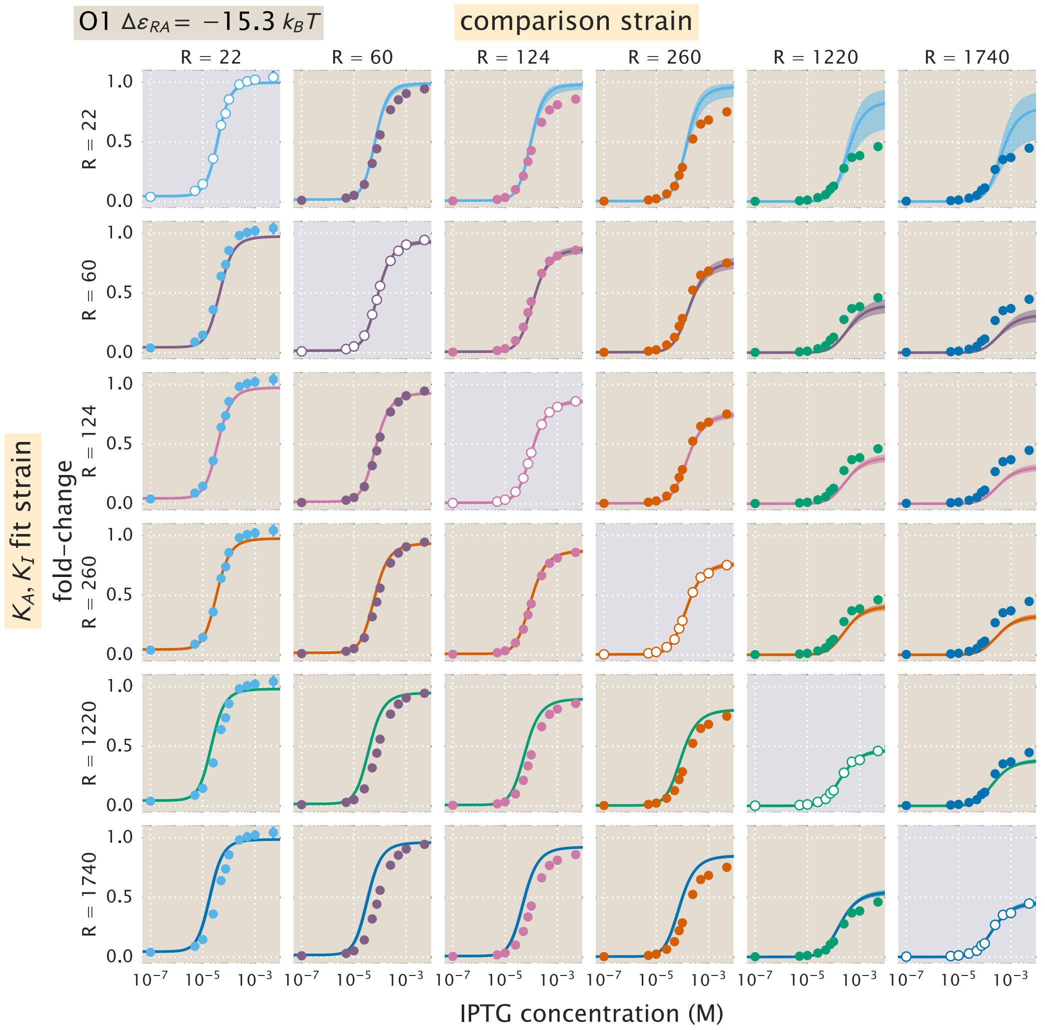

Figure S11. O1 strain fold-change predictions based on strain-specific parameter estimation of K_A and K_I, related to STAR Methods. Fold-change in expression is plotted as a function of IPTG concentration for all strains containing an O1 operator. The solid points correspond to the mean experimental value. The solid lines correspond to Eq. (5) using the parameter estimates of K_A and K_I. Each row uses a single set of parameter values based on the strain noted on the left axis. The shaded plots along the diagonal are those where the parameter estimates are plotted along with the data used to infer them. Values for repressor copy number and operator binding energy are from Garcia and Phillips (2011a). The shaded region on the curve represents the uncertainty from our parameter estimates and reflect the 95% highest probability density region of the parameter predictions.

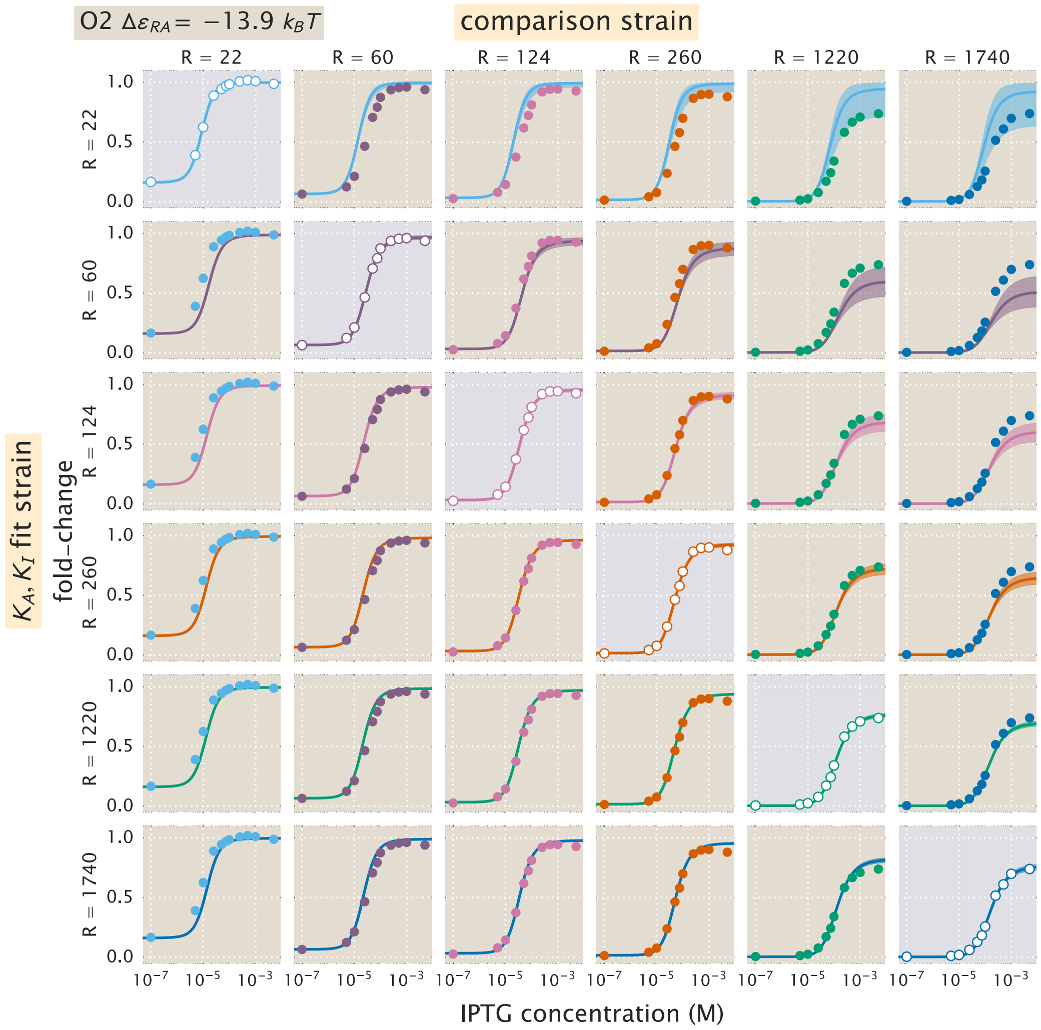

Figure S12. O2 strain fold-change predictions based on strain-specific parameter estimation of K_A and K_I, related to STAR Methods. Fold-change in expression is plotted as a function of IPTG concentration for all strains containing an O2 operator. The plots and data shown are analogous to Fig. S11, but for the O2 operator.

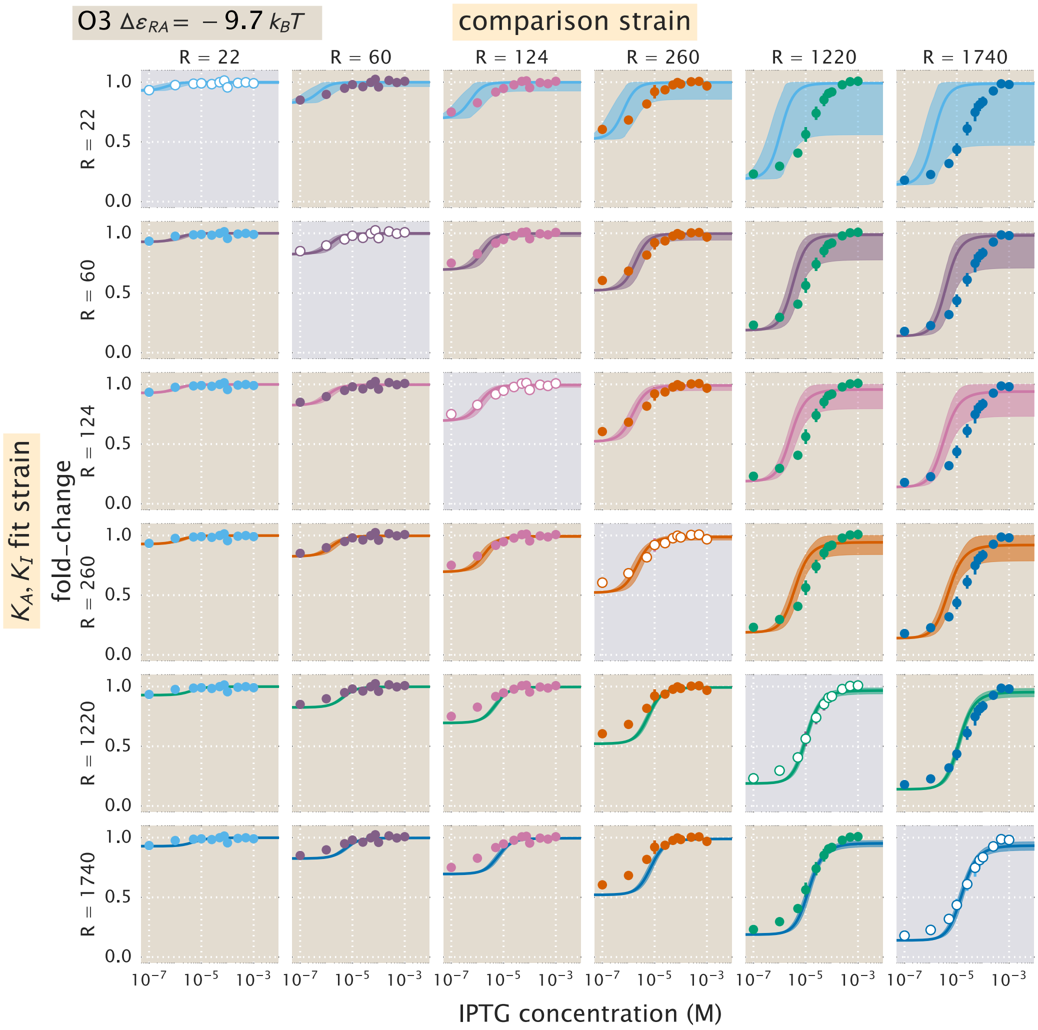

Figure S13. O3 strain fold-change predictions based on strain-specific parameter estimation of K_A and K_I, related to STAR Methods. Fold-change in expression is plotted as a function of IPTG concentration for all strains containing an O3 operator. The plots and data shown are analogous to Fig. S11, but for the O3 operator. We note that when using the $R = 22$ O3 strain to predict K_A and K_I, the large uncertainty in the estimates of these parameters (see Fig. 5(D)) leads to correspondingly wider credible regions.

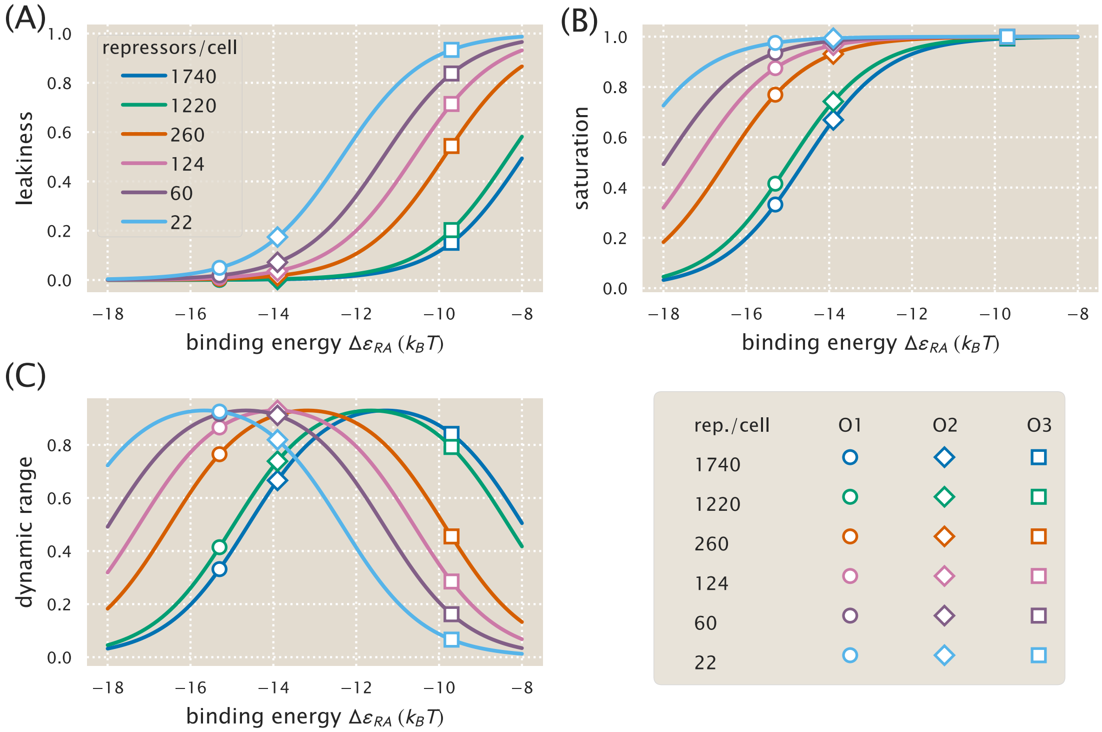

Figure S14. Dependence of leakiness, saturation, and dynamic range on the operator binding energy and repressor copy number, related to STAR Methods. Increasing repressor copy number or decreasing the repressor-operator binding energy suppresses gene expression and decreases both the (A) leakiness and (B) saturation. (C) The dynamic range retains its shape but shifts right as the repressor copy number increases. The peak in the dynamic range can be understood by considering the two extremes for $\Delta\varepsilon_{RA}$: for small repressor-operator binding energies, the leakiness is small but the saturation increases with $\Delta\varepsilon_{RA}$; for large repressor-operator binding energies the saturation is near unity and the leakiness increases with $\Delta\varepsilon_{RA}$, thereby decreasing the dynamic range. Repressor copy number does not affect the maximum dynamic range (see Eq. (21)). Circles, diamonds, and squares represent $\Delta\varepsilon_{RA}$ values for the O1, O2, and O3 operators, respectively, demonstrating the expected values of the properties using those strains.

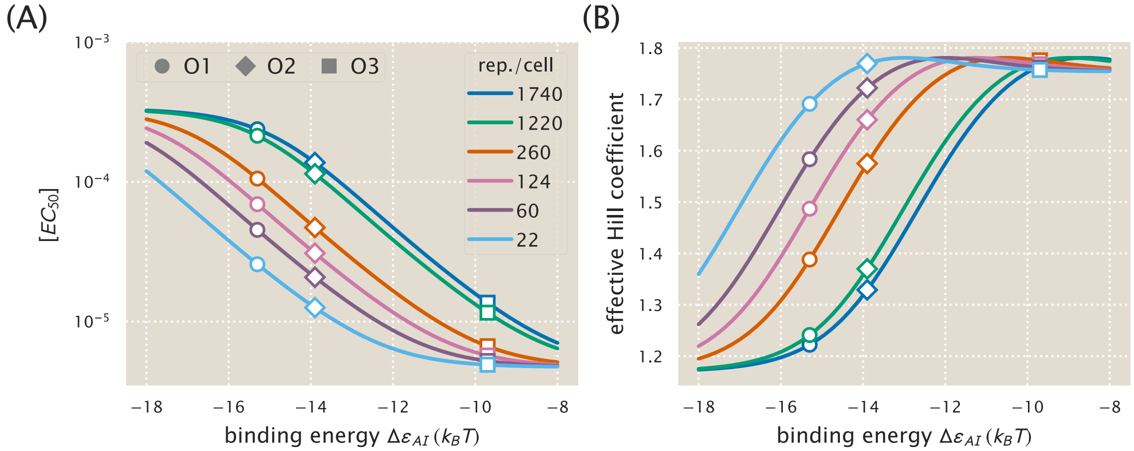

Figure S15. [EC_{50}] and effective Hill coefficient depend strongly on repressor copy number and operator binding energy, related to STAR Methods. (A) [EC_{50}] values range from very small and tightly clustered at weak operator binding energies (e.g. O3) to relatively large and spread out for stronger operator binding energies (O1 and O2). (B) The effective Hill coefficient generally decreases with increasing repressor copy number, indicating a flatter normalized response. The maximum possible Hill coefficient is roughly 1.75 for all repressor-operator binding energies. Circles, diamonds, and squares represent $\Delta\varepsilon_{RA}$ values for the O1, O2, and O3 operators, respectively.

Table S1. Primers used in this work, related to STAR Methods. Lower case sequences denote homology to a chromosomal locus used for integration of the construct into the *E. coli* chromosome. Uppercase sequences refer to the sequences used for PCR amplification.

**Primer**	**Sequence**	**Comment**
General sequencing primers:		
pZSForwSeq2	TTCCCAACCTTACCAGAGGGC	Forward primer for 3*1x-lacI
251F	CCTTTCGTCTTCACCTCGA	Forward primer for 25x+11-yfp
YFP1	ACTAGCAACACCAGAACAGCCC	Reverse primer for 3*1x-lacI and 25x+11-yfp
Integration primers:		
HG6.1 (*galK*)	gtttgcgcgcagtcagcgatatccattttcgcgaatccgg agtgtaagaaACTAGCAACACCAGAACAGCC	Reverse primer for 25x+11-yfp with homology to *galK* locus.
HG6.3 (*galK*)	ttcatattgttcagcgacagcttgctgtacggcaggcacc agctcttccgGGCTAATGCACCCAGTAAGG	Forward primer for 25x+11-yfp with homology to *galK* locus.
galK-control-upstream1	TTCATATTGTTCAGCGACAGCTTG	To check integration.
galK-control-downstream1	CTCCGCCACCGTACGTAAATT	To check integration.
HG11.1 (*ybcN*)	acctctgcggaggggaagcgtgaacctctcacaagacggc atcaaattacACTAGCAACACCAGAACAGCC	Reverse primer for 3*1x-lacI with homology to *ybcN* locus.
HG11.3 (*ybcN*)	ctgtagatgtgtccgttcatgacacgaataagcggtgtag ccattacgccGGCTAATGCACCCAGTAAGG	Forward primer for 3*1x-lacI with homology to *ybcN* locus.
ybcN-control-upstream1	AGCGTTTGACCTCTGCGGA	To check integration.
ybcN-control-downstream1	GCTCAGGTTTACGCTTACGACG	To check integration.

Table S2. *E. coli* strains used in this work, related to STAR Methods. Each strain contains a unique operator-yfp construct for measurement of fluorescence and R refers to the dimer copy number as measured by Garcia and Phillips (2011a).

Strain	Genotype
O1, $R = 0$	HG105::galK⟨⟩25O1+11-yfp
O1, $R = 22$	HG104::galK⟨⟩25O1+11-yfp
O1, $R = 60$	HG105::galK⟨⟩25O1+11-yfp, ybcN⟨⟩3*1RBS1147-lacI
O1, $R = 124$	HG105::galK⟨⟩25O1+11-yfp, ybcN⟨⟩3*1RBS1027-lacI
O1, $R = 260$	HG105::galK⟨⟩25O1+11-yfp, ybcN⟨⟩3*1RBS446-lacI
O1, $R = 1220$	HG105::galK⟨⟩25O1+11-yfp, ybcN⟨⟩3*1RBS1-lacI
O1, $R = 1740$	HG105::galK⟨⟩25O1+11-yfp, ybcN⟨⟩3*1-lacI (RBS1L)
O2, $R = 0$	HG105::galK⟨⟩25O2+11-yfp
O2, $R = 22$	HG104::galK⟨⟩25O2+11-yfp
O2, $R = 60$	HG105::galK⟨⟩25O2+11-yfp, ybcN⟨⟩3*1RBS1147-lacI
O2, $R = 124$	HG105::galK⟨⟩25O2+11-yfp, ybcN⟨⟩3*1RBS1027-lacI
O2, $R = 260$	HG105::galK⟨⟩25O2+11-yfp, ybcN⟨⟩3*1RBS446-lacI
O2, $R = 1220$	HG105::galK⟨⟩25O2+11-yfp, ybcN⟨⟩3*1RBS1-lacI
O2, $R = 1740$	HG105::galK⟨⟩25O2+11-yfp, ybcN⟨⟩3*1-lacI (RBS1L)
O3, $R = 0$	HG105::galK⟨⟩25O3+11-yfp
O3, $R = 22$	HG104::galK⟨⟩25O3+11-yfp
O3, $R = 60$	HG105::galK⟨⟩25O3+11-yfp, ybcN⟨⟩3*1RBS1147-lacI
O3, $R = 124$	HG105::galK⟨⟩25O3+11-yfp, ybcN⟨⟩3*1RBS1027-lacI
O3, $R = 260$	HG105::galK⟨⟩25O3+11-yfp, ybcN⟨⟩3*1RBS446-lacI
O3, $R = 1220$	HG105::galK⟨⟩25O3+11-yfp, ybcN⟨⟩3*1RBS1-lacI
O3, $R = 1740$	HG105::galK⟨⟩25O3+11-yfp, ybcN⟨⟩3*1-lacI (RBS1L)
Oid, $R = 0$	HG105::galK⟨⟩25Oid+11-yfp
Oid, $R = 22$	HG104::galK⟨⟩25Oid+11-yfp
Oid, $R = 60$	HG105::galK⟨⟩25Oid+11-yfp, ybcN⟨⟩3*1RBS1147-lacI
Oid, $R = 124$	HG105::galK⟨⟩25Oid+11-yfp, ybcN⟨⟩3*1RBS1027-lacI
Oid, $R = 260$	HG105::galK⟨⟩25Oid+11-yfp, ybcN⟨⟩3*1RBS446-lacI
Oid, $R = 1220$	HG105::galK⟨⟩25Oid+11-yfp, ybcN⟨⟩3*1RBS1-lacI
Oid, $R = 1740$	HG105::galK⟨⟩25Oid+11-yfp, ybcN⟨⟩3*1-lacI (RBS1L)

Table S3. Instrument settings for data collection using the Miltenyi Biotec MACSQuant flow cytometer, related to STAR Methods. All experimental measurements were collected using these values.

Laser	Channel	Sensor Voltage
488 nm	Forward-Scatter (FSC)	423 V
488 nm	Side-Scatter (SSC)	537 V
488 nm	Intensity (B1 Filter, 525/50nm)	790 V
488 nm	Trigger (debris threshold)	24.5 V

Table S4. Key model parameters for induction of an allosteric repressor, related to STAR Methods.

Parameter	Description
c	Concentration of the inducer
K_A, K_I	Dissociation constant between an inducer and the repressor in the active/inactive state
$\Delta\varepsilon_{AI}$	The difference between the free energy of repressor in the inactive and active states
$\Delta\varepsilon_P$	Binding energy between the RNAP and its specific binding site
$\Delta\varepsilon_{RA}, \Delta\varepsilon_{RI}$	Binding energy between the operator and the active/inactive repressor
n	Number of inducer binding sites per repressor
P	Number of RNAP
R_A, R_I, R	Number of active/inactive/total repressors
$p_A = \frac{R_A}{R}$	Probability that a repressor will be in the active state
p_{bound}	Probability that an RNAP is bound to the promoter of interest, assumed to be proportional to gene expression
fold-change	Ratio of gene expression in the presence of repressor to that in the absence of repressor
F	Free energy of the system
N_{NS}	The number of non-specific binding sites for the repressor in the genome
$\beta = \frac{1}{k_B T}$	The inverse product of the Boltzmann constant k_B and the temperature T of the system

Table S5. Global fit of all parameter values using the entire data set. Related to Figure 5 and STAR Methods. In addition to fitting the repressor inducer dissociation constants K_A and K_I as was done in the text, we also fit the repressor DNA binding energy $\Delta\varepsilon_{RA}$ as well as the repressor copy numbers R for each strain. The middle columns show the previously reported values for all $\Delta\varepsilon_{RA}$ and R values, with $\pm$ representing the standard deviation of three replicates. The right column shows the global fits from this work, with the subscript and superscript notation denoting the 95% credible region. Note that there is overlap between all of the repressor copy numbers and that the net difference in the repressor-DNA binding energies is less than 1 k_BT. The logarithms $\tilde{k}_A = -\log \frac{K_A}{1\,\mathrm{M}}$ and $\tilde{k}_I = -\log \frac{K_I}{1\,\mathrm{M}}$ of the dissociation constants were fit for numerical stability.

	Reported Values (Garcia and Phillips 2011a)	Global Fit
$\tilde{k}_A$	–	$-5.33^{+0.06}_{-0.05}$
$\tilde{k}_I$	–	$0.31^{+0.05}_{-0.06}$
K_A	–	$205^{+11}_{-12}\,\mu$M
K_I	–	$0.73^{+0.04}_{-0.04}\,\mu$M
R_{22}	22 ± 4	20^{+1}_{-1}
R_{60}	60 ± 20	74^{+4}_{-3}
R_{124}	124 ± 30	130^{+6}_{-6}
R_{260}	260 ± 40	257^{+9}_{-11}
R_{1220}	1220 ± 160	1191^{+32}_{-55}
R_{1740}	1740 ± 340	1599^{+75}_{-87}
O1 $\Delta\varepsilon_{RA}$	$-15.3 \pm 0.2\ k_BT$	$-15.2^{+0.1}_{-0.1}\ k_BT$
O2 $\Delta\varepsilon_{RA}$	$-13.9 \pm 0.2\ k_BT$	$-13.6^{+0.1}_{-0.1}\ k_BT$
O3 $\Delta\varepsilon_{RA}$	$-9.7 \pm 0.1\ k_BT$	$-9.4^{+0.1}_{-0.1}\ k_BT$
Oid $\Delta\varepsilon_{RA}$	$-17.0 \pm 0.2\ k_BT$	$-17.7^{+0.2}_{-0.1}\ k_BT$
